# Structure, thermal expansion and incompressibility of MgSO_4_·9H_2_O, its relationship to meridianiite (MgSO_4_·11H_2_O) and possible natural occurrences

**DOI:** 10.1107/S2052520616018266

**Published:** 2017-01-31

**Authors:** A. Dominic Fortes, Kevin S. Knight, Ian G. Wood

**Affiliations:** a ISIS Facility, STFC Rutherford Appleton Laboratory, Harwell Science and Innovation Campus, Chilton, Oxfordshire OX11 0QX, England; b Department of Earth Sciences, University College London, Gower Street, London WC1E 6BT, England; c The Natural History Museum, Cromwell Road, London SW7 5BD, England

**Keywords:** enneahydrate, meridianiite, undecahydrate, thermal expansion, equation of state, neutron diffraction, DFT, elastic constants

## Abstract

Since being discovered initially in mixed-cation systems, a method of forming end-member MgSO_4_·9H_2_O has been found. We have obtained powder diffraction data from protonated analogues (using X-rays) and deuterated analogues (using neutrons) of this compound over a range of temperatures and pressures. From these data we have determined the crystal structure, including all hydrogen positions, the thermal expansion over the range 9–260 K at ambient pressure, the incompressibility over the range 0–1.1 GPa at 240 K and studied the transitions to other stable and metastable phases. MgSO_4_·9D_2_O is monoclinic, space group *P*2_1_/*c*, *Z* = 4, with unit-cell parameters at 9 K, *a* = 6.72764 (6), *b* = 11.91154 (9), *c* = 14.6424 (1) Å, β = 95.2046 (7)° and *V* = 1168.55 (1) Å^3^. The structure consists of two symmetry-inequivalent Mg(D_2_O)_6_ octahedra on sites of  symmetry. These are directly joined by a water–water hydrogen bond to form chains of octahedra parallel with the *b* axis at *a* = 0. Three interstitial water molecules bridge the Mg(D_2_O)_6_ octahedra to the SO_4_^2−^ tetrahedral oxyanion. These tetrahedra sit at *a* ≃ 0.5 and are linked by two of the three interstitial water molecules in a pentagonal motif to form ribbons parallel with *b*. The temperature dependences of the lattice parameters from 9 to 260 K have been fitted with a modified Einstein oscillator model, which was used to obtain the coefficients of the thermal expansion tensor. The volume thermal expansion coefficient, α_V_, is substantially larger than that of either MgSO_4_·7D_2_O (epsomite) or MgSO_4_·11D_2_O (meridianiite), being ∼ 110 × 10^−6^ K^−1^ at 240 K. Fitting to a Murnaghan integrated linear equation of state gave a zero-pressure bulk modulus for MgSO_4_·9D_2_O at 240 K, *K*_0_ = 19.5 (3) GPa, with the first pressure derivative of the bulk modulus, *K*′ = 3.8 (4). The bulk modulus is virtually identical to meridianiite and only ∼ 14% smaller than that of epsomite. Above ∼ 1 GPa at 240 K the bulk modulus begins to decrease with pressure; this elastic softening may indicate a phase transition at a pressure above ∼ 2 GPa. Synthesis of MgSO_4_·9H_2_O from cation-pure aqueous solutions requires quench-freezing of small droplets, a situation that may be relevant to spraying of MgSO_4_-rich cryomagmas into the surface environments of icy satellites in the outer solar system. However, serendipitously, we obtained a mixture of MgSO_4_·9H_2_O, mirabilite (Na_2_SO_4_·10H_2_O) and ice by simply leaving a bottle of mid-winter brine from Spotted Lake (Mg/Na ratio = 3), British Columbia, in a domestic freezer for a few hours. This suggests that MgSO_4_·9H_2_O can occur naturally – albeit on a transient basis – in certain terrestrial and extraterrestrial environments.

## Abstract 2

We have employed neutron and X-ray powder diffraction and density functional theory calculations to determine the structure and thermoelastic properties of a new hydrate in the MgSO_4_–H_2_O binary system, magnesium sulfate enneahydrate. We show that this 9-hydrate could occur naturally in certain hypersaline lakes on Earth and indicate where it may be formed as a more persistent mineral elsewhere in the solar system.

## 1. Introduction   

### 1.1. Background   

Crystalline hydrates of the general form MgSO_4_·*n*H_2_O with *n* = 1, 1.25, 2, 2.5, 3, 4, 5, 6, 7 and 11 have been known for over a century and their ranges of stability and metastability are reasonably well established (D’Ans, 1933 ▸; Hodenberg & Kühn, 1967 ▸). The lack of any analogues with *n* = 11 in other divalent metal sulfates, and the curious gap in hydration states between *n* = 7 and *n* = 11 led us to investigate methods of synthesizing hitherto unknown compounds and new hydration states. Our attention was originally drawn to this gap between *n* = 7 and *n* = 11 by high-pressure studies of MgSO_4_·11H_2_O where we observed an apparent decomposition at ∼ 0.9 GPa, 240 K to a mixture of ice VI and what was – at the time – an unknown hydrate (Fortes *et al.*, 2017 ▸). The unidentified phase did not correspond to any of the high-pressure phases of MgSO_4_·7D_2_O we had encountered in other high-pressure experiments (Gromnitskaya *et al.*, 2013 ▸) and one possible explanation was the occurrence of an intermediate hydration state that became stable only under pressure.

In our experiments we initially adopted chemical substitution as a crude proxy for hydrostatic stress, the expectation being that this would create analogue structures of the phase observed at high pressure. The work paid dividends in terms of characterizing the extent to which various divalent metal cations can substitute for Mg^2+^ in the meridianiite structure and their effect on the unit-cell parameters (Fortes, Browning & Wood, 2012*a* ▸,*b* ▸), and also revealed crystals isotypic with meridianiite formed from MgCrO_4_ and MgSeO_4_ (Fortes & Wood, 2012 ▸: Fortes, 2015 ▸). Furthermore, by substituting Mg^2+^ with Zn^2+^, Ni^2+^ or Fe^2+^, we determined that novel hydration states could indeed be formed in mixed-cation compounds [*i.e.* (Mg_1 − *x*_Ni*_x_*)SO_4_ for 0.3 < *x* < 0.8] by rapid quenching of aqueous solutions in liquid nitrogen. These included a monoclinic enneahydrate for all of the aforementioned cations and a triclinic octahydrate for Ni^2+^. The discovery of MgSO_4_·9H_2_O also resolved a long-standing question over the origin of weak parasitic peaks observed in our first neutron powder diffraction measurement on MgSO_4_·11D_2_O, which did not appear to match any other known hydrate or plausible contaminant (Fortes *et al.*, 2008 ▸, and Fig. S1 of the supporting information).

Following its identification, the new enneahydrate became our best candidate for the phase that forms when meridianiite decomposes at high pressure, implying that MgSO_4_·9H_2_O might occur naturally at depth inside the largest icy satellites of Jupiter, where MgSO_4_/Na_2_SO_4_-brine oceans are believed to be present (Khurana *et al.*, 1998 ▸; Kivelson *et al.*, 2002 ▸; Vance & Brown, 2013 ▸; Vance *et al.*, 2014 ▸) and where MgSO_4_ and Na_2_SO_4_ hydrates have been reported in surface deposits from near-IR spectroscopy (Orlando *et al.*, 2005 ▸; Dalton *et al.*, 2005 ▸; Shirley *et al.*, 2010 ▸). Since it may be a ‘rock-forming’ mineral in the outer solar system, and since we have also found it in slowly frozen natural brine (see §4), this compound and its relationship to other cryohydrates acquires mineralogical significance.

For brevity, the composition of these phases will be written in the form MS*n*, where M = Mg, S = SO_4_ and *n* identifies the number of water molecules per formula unit. Hence, MS9 would mean MgSO_4_·9H_2_O, MS11 = MgSO_4_·11H_2_O and so on. The addition of ‘(D)’ indicates a deuterated analogue, such that MS9(D) means MgSO_4_·9D_2_O.

### 1.2. Experimental objectives   

The objectives of this work are: (i) to determine the complete structure of MS9, including all hydrogen positions for comparison with the recently determined structure of MgSeO_4_·9H_2_O (Fortes *et al.*, 2015 ▸); (ii) to determine the unit-cell parameters of MS9 as a function of temperature and pressure in order to characterize the thermal expansion and compressibility of the structure; (iii) to obtain data suitable for testing our hypothesis that MS11 decomposes to MS9 + ice at high pressure. We have used a combination of techniques to achieve these goals, including X-ray and neutron powder diffraction and density functional theory (DFT) calculations. A necessary consequence of using neutron scattering is the requirement to employ a deuterated analogue, MS9(D), in order to eliminate the undesirable incoherent neutron scattering from ^1^H. Although there are precedents where materials exhibit substantially different phase behaviour on deuteration, our experience of working with many different forms of ice and salt hydrates is that deuteration has a negligible effect on the molar volume and shifts by just a few percent the values of thermoelastic parameters and locations of phase boundaries (*e.g.* Pistorius, 1968 ▸; Fortes, Wood, Tucker & Marshall, 2012 ▸).

This work forms the middle part of a series of three papers. The first deals with the high-pressure behaviour of meridianiite (MS11) and its decomposition to the enneahydrate: the third paper, which will be presented elsewhere, deals with the Ni-bearing species, NiSO_4_·9H_2_O and its solid solution series with MS9, and with the structure of NiSO_4_·8H_2_O.

## 2. Experimental method   

### 2.1. Sample synthesis   

Aqueous solutions were prepared from MgSO_4_ (Sigma-Aldrich M7506, anhydrous β-phase, *ReagentPlus* ^®^, ≥ 99.5%) in either H_2_O (Alfa-Aesar, ACS Reagent Grade, 36645) or D_2_O (Aldrich 151882 99.9 atom % D). We endeavoured to prepare the most concentrated solutions possible, since this reduces the amount of accessory ice in the final sample. However, highly supersaturated solutions of either MgSO_4_ tend to be quite labile, crystallizing very quickly, so the typical concentrations used here are in the region of 30 wt % dissolved solids, yielding mixtures containing ∼ 70 wt % MS9 and 30 wt % water ice. Once it became clear how deleterious the presence of ice in the sample can be, efforts were made to synthesize and flash-freeze a starting solution with the same stoichiometry as the 9-hydrate itself, *i.e.* 42.6 wt % MgSO_4_. Such a solution may be prepared by very slow evaporation, without agitation, of an initially undersaturated solution (*e.g.* 25 wt %) at a temperature of 373–383 K. Ordinarily, this process will lead to the crystallization of metastable MS3, but rapid quenching of the solution prior to the appearance of trihydrate crystals can produce a virtually phase pure specimen of the enneahydrate.

Droplets were pipetted directly into liquid nitrogen held in a steel cryomortar, producing glassy solid spherules in the region of 2–4 mm in diameter. The amorphous spherules are pulverized with a nitrogen-chilled steel pestle and then transferred to an appropriate sample holder. This work was carried in the UCL Earth Sciences Cold Rooms at an air temperature of around 261 K so as to minimize the incorporation of ice formed from atmospheric water vapour into the sample.

### 2.2. X-ray powder diffraction   

X-ray powder diffraction data were obtained in Bragg–Brentano parafocussing reflection geometry using a PAN­analytical X’Pert Pro multipurpose powder diffractometer with germanium-monochromated Co *K*α_1_ radiation (λ = 1.788996 Å, and an X’Celerator multi-strip detector). Data were collected with variable divergence and receiving slits, converted to fixed-slit geometry with the proprietary *X’Pert Pro HighScore Plus* software package, and exported in an appropriate format for analysis in the *GSAS/Expgui* package (Larsen & Von Dreele, 2000 ▸; Toby, 2001 ▸).

Stable low-temperature measurements were achieved using a thermoelectrically cooled cold stage (Wood *et al.*, 2012 ▸). This portable cold stage was held in a plastic box filled with dry-ice pellets whilst the powder specimen was prepared and loaded. Powder samples were transferred to the cold stage and packed down using a liquid nitrogen-cooled spatula to form a top-loaded pressed-powder specimen. A cover with an X-ray transparent window was screwed into place, which preserves a cold and dry atmosphere over the specimen during mounting on the diffractometer and subsequent measurement. The presence of a substantial open space (∼ 52 cm^3^) over the sample and beneath the cover, which is not actively cooled, means that there is scope for sublimation of ice and transport of vapour over the top of the specimen. In early studies of ice-rich enneahydrate samples, we found that the MS9 would transform irreversibly to the more stable undecahydrate, MS11, on time scales of hours at 250 K, presumably by reaction with water vapour. This motivated our efforts to synthesize ice-free samples, simply in order to allow for measurements over a large angular range with little background noise without the specimen transforming to another phase.

### 2.3. Neutron powder diffraction (ISIS)   

Neutron powder diffraction data were collected using the high-resolution powder diffractometer (HRPD; Ibberson *et al.*, 1992 ▸; Ibberson, 2009 ▸) at the STFC ISIS spallation neutron source, Rutherford Appleton Laboratory, UK. For ambient-pressure analysis, the powdered samples were transferred directly into a pre-cooled sample holder mounted on a cryostat centre stick. The sample holder consisted of an aluminium holder with a slab-geometry space of dimensions (relative to the incident beam) of width = 18 mm, height = 23 mm and depth = 15 mm. Steel-framed vanadium windows were screwed to the front and back of the holder, the exposed Al and steel components on the front face being masked by Gd foil.

Samples were mounted in the cryostat at 100–150 K, where they were observed to be amorphous. Slow incremental warming to 230 K resulted in crystallization, after which the temperature was reduced to 10 K to collect powder diffraction data suitable for structure refinement.

#### 2.3.1. Cation-pure MgSO_4_ samples at ambient pressure   

An attempt was made initially to synthesize phase-pure MS9(D), which resulted in a degree of disproportionation and formation of a sample containing substantial MS11(D) and some water ice, the latter exhibiting rather broad Bragg peaks. Despite the sub-optimal quality of the specimen, data were collected at base temperature and in 5 K increments on warming back up to 230 K using the long time window (100–200 ms time-of-flight) on HRPD; in backscattering this provides high-resolution data covering *d*-spacings from 2.2 to 4 Å. This dataset is referred to as Series 1.

Subsequently, a second sample was prepared with a more water-rich stoichiometry. As before, this was loaded at 150 K and crystallized after warming to 220 K. Inevitably, this sample contained more water ice, but the co-existing material was phase-pure 9-hydrate and all Bragg peaks were sharper than those in Series 1. Powder diffraction data were obtained on warming in 5 K increments from 220 K to 265 K using the 100–200 ms time window. These data (Series 2) recorded the onset of MS9(D) transforming to MS11(D) at 250 K, a process that was complete upon reaching 265 K on the *ca* 1 h timescale of each measurement.

A third specimen consisting, like the second, of ∼ 70 wt % MS9(D) and 30 wt % deuterated ice Ih, was prepared in the same fashion and cooled to 9 K where diffraction data were acquired in both the short and long time windows, 30–130 ms and 100–200 ms, for ∼ 17 and 13 h, respectively. Data were then collected in the 100–200 ms window on warming to 220 K in 10 K increments. In order to evaluate the transformation of the 9-hydrate to the 11-hydrate, the sample was warmed first from 220 to 250 K and data were collected in 20 min snapshots for ∼ 2 h, and then up to 260 K with 20 min shots being acquired sequentially for ∼ 5.5 h. This dataset comprises Series 3.

#### 2.3.2. Cation-pure MgSO_4_ samples at non-ambient pressure   

A fourth aliquot of flash-frozen material was loaded into a TiZr gas-pressure vessel embedded in dry-ice snow. Crystallization occurred as the sample was warmed from 220 to 240 K under 10 MPa of He gas to a mixture dominated by MS9(D) with ∼ 25 wt % MS7(D). Data were collected in the 30–130 ms time window in HRPD’s 90° detector banks, which have a larger solid angle coverage than the backscattering detectors but a lower resolution (Δ*d*/*d* ≃ 2 × 10^−3^). Using He gas as the pressure-transmitting medium, diffraction patterns were acquired in roughly 100 MPa increments along the 240 K isotherm up to ∼ 540 MPa with counting times of ∼ 12 h each. The unmasked pressure vessel contributes a number of parasitic features to the diffraction pattern, as well as a substantial *Q*-dependent background, which was corrected by subtraction of an empty-cell measurement collected at the end of the experiment.

### 2.4. *Ab initio* calculations   

In order to aid in the interpretation of some of our experimental data, and to predict certain quantities we are unable to measure, we carried out a series of first-principles calculations using DFT and the plane-wave pseudopotential method (Hohenberg & Kohn, 1964 ▸; Kohn & Sham, 1965 ▸). The calculations were carried out using *CASTEP* (Payne *et al.*, 1992 ▸; Segall *et al.*, 2002 ▸; Clark *et al.*, 2005 ▸) in conjunction with the analysis tools in the *Materials Studio* software package (http://accelrys.com). A basis-set cut-off of 1200 eV and a 4 × 2 × 2 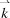-point grid (∼ 0.04 Å^−1^ reciprocal lattice spacing) were required to achieve convergence of better than 1 × 10^−2^ GPa in the stress and better than 1 × 10^−4^ eV per atom in total energy. We also tested two different gradient-corrected functionals (GGAs), specifically those referred to as ‘PBE’ (Perdew *et al.*, 1996 ▸, 1997 ▸) and ‘Wu–Cohen’ or ‘WC’ (Wu & Cohen, 2006 ▸). In the past, we have successfully used the PW91 or closely related PBE GGAs for these types of materials (Fortes *et al.*, 2014 ▸, 2015 ▸). However, there is reason to believe that the Wu–Cohen GGA functional is more accurate than PBE for specific types of calculations (*e.g.* Tran *et al.*, 2007 ▸; Haas *et al.*, 2009 ▸, 2011 ▸), correcting the under-binding commonly encountered in PW91 and PBE simulations.

Structural relaxations under zero-pressure athermal conditions were carried out, starting from the experimental crystal structure obtained at 9 K for MS9 (see §3.1), using the BFGS method (Pfrommer *et al.*, 1997 ▸). The relaxations were considered to have converged when the forces on each atom were less than 5 × 10^−3^ eV Å^−1^ and each component of the stress tensor was smaller than 0.005 GPa. Details of how other specific properties were calculated are given in subsequent sections; the exception to this is the calculation of the elastic constants, which appears in the supporting information.

## 3. Results   

### 3.1. Structure determination and completion   

From prior work (Fortes, Browning & Wood, 2012*a* ▸,*b* ▸) it was known that MS9 is monoclinic, with unit-cell parameters at 250 K *a* = 6.75 Å, *b* = 11.95 Å, *c* = 14.65 Å, β = 95.1°, *V* = 1177 Å^3^ with *Z* = 4. Systematic absences indicated that the most probable space group was *P*2_1_/*c*; the space group *Pc* remained a possibility due to the limited number of well separated *k*-odd 0*k*0 reflections observable by eye in the low-angle portion of the pattern and since Pawley refinements in both space groups only weakly favoured *P*2_1_/*c*. We also observed that in X-ray powder data cation-pure MS9 produces 100 peaks of measurable intensity, whilst the 011 peak is of zero (or negligible) intensity. However, in Ni-doped samples of MS9 the pattern is reversed, 011 is present and 100 has no measureable intensity (Fig. S2). We interpreted this as being due to the preferential occupancy of particular Mg^2+^ sites in the structure by Ni^2+^, which requires there to be at least two symmetry-independent octahedral sites. This is possible in the space group *Pc* with the octahedrally coordinated cations on general positions; should the space group be *P*2_1_/*c*, then this obliges the cations to sit on special positions and these sites (2*a* and 2*c*) occupy separate 011 planes, thus permitting site ordering.

In an effort to avoid unconscious bias in the structure solution process concerning either the space group or the siting of Mg(H_2_O)_6_ octahedra on general or special positions, the structure was solved initially in the space group *Pc*. Since the earlier identification of this phase as a 9-hydrate was based purely on unit-cell volume considerations, we also chose to ‘test’ the stoichiometry by solving the structure without any interstitial water and then evaluating difference Fourier maps phased on any partial solution. X-ray powder data obtained by quenching a highly concentrated solution, which contained largely MS9 and only a very small amount of MS11 (< 5 wt %), were used as the basis for the structure solution, which was achieved using the parallel tempering algorithm in *FOX*, Version 1.9.7.1 (Favre-Nicolin & Černý, 2002 ▸, 2004 ▸). For the 9-hydrate, FOX was used to construct ideal MgO_6_ octahedra with Mg—O distances of 2.08 Å, and ideal SO_4_ tetrahedra with S—O distances of 1.48 Å; these were treated as rigid bodies throughout the solution process. In runs of half a million trials each, the crystal structure was optimized against the powder diffraction data, consistently producing very similar structures with chemically sensible arrangements of the ionic polyhedra even after only a few 10 s of thousands of trials. Difference Fourier maps phased on these structures revealed three peaks per formula unit that correspond to the additional water molecules necessary to form a 9-hydrate (Fig. S3). Ultimately the structure with the lowest overall cost function was exported as a CIF file to form the basis for further analysis.

Examination of the most favourable solution revealed that the cations occupied centres of symmetry; consequently, the true space group must indeed be *P*2_1_/*c*, as expected, with Mg^2+^ ions occupying the 2*a* and 2*c* special positions (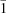 site symmetry) and so the structure was transformed into the higher-symmetry setting.

The trial heavy-atom structure was refined against the X-ray powder dataset using GSAS/Expgui with quite stiff bond distance and bond angle restraints, Mg—O bond lengths being restrained to be 2.08 (4) Å, S—O bond lengths being restrained to 1.480 (5) Å, and the SO_4_^2−^ oxyanion being forced to adopt ideal tetrahedral symmetry (O—S—O angles = 109.5°). Study of the first coordination shell of the water O atoms at the end of this refinement revealed a sensible pattern of O⋯O vectors of length 2.7–3.0 Å, consistent with hydrogen-bonded contacts, and so pairs of H atoms were sited at distances of 0.98 Å from each O atom along these vectors.

The complete structure was then refined against the 9 K neutron powder diffraction data for MS9(D) (Series 3). Since these data, particularly at short *d*-spacings, are dominated by Bragg peaks from water ice, it was necessary to apply similar bond distance and angle restraints to those mentioned earlier and to constrain isotropic displacement parameters for light/heavy atoms to shift together. In the later stages of the refinements, restraint weightings were removed.

**Figure 1:**
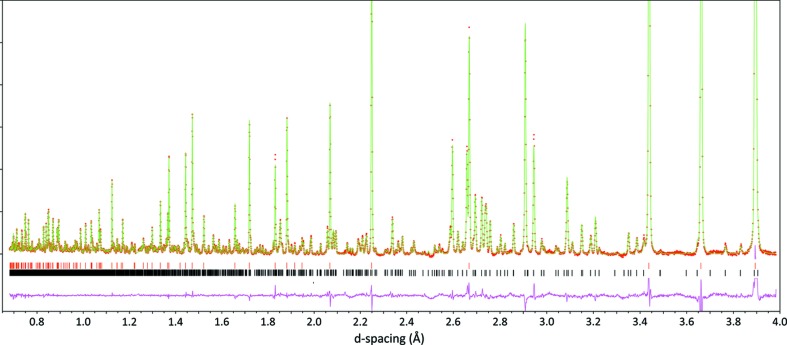
Rietveld refinement of the MgSO_4_·9D_2_O structure against neutron powder diffraction data obtained at 9 K in HRPD’s 30–130 ms window and 100–200 ms window. The graphical output for each histogram’s refinement has been stitched together at *d* = 2.4 Å. Filled red circles report the observed data, solid green lines the calculated fit and the purple line underneath is obs − calc. Note that, whilst MS9 represents almost 50% by weight of the sample, its larger unit-cell and lower symmetry mean that the intensity of the strongest MS9 peaks is many times less than water ice; consequently, the vertical scale has been greatly expanded, with the effect that the difference curve – particularly in the vicinity of the ice peaks – appears noisy. Tick marks for water ice are uppermost and those for MgSO_4_·9D_2_O are below them.

A graphical representation of the fit is given in Fig. 1 ▸, structural parameters are given in the supporting CIF data and selected bond distances and angles are reported in Tables 1 ▸ and 2 ▸ for comparison with the DFT-derived values.

**Figure 2:**
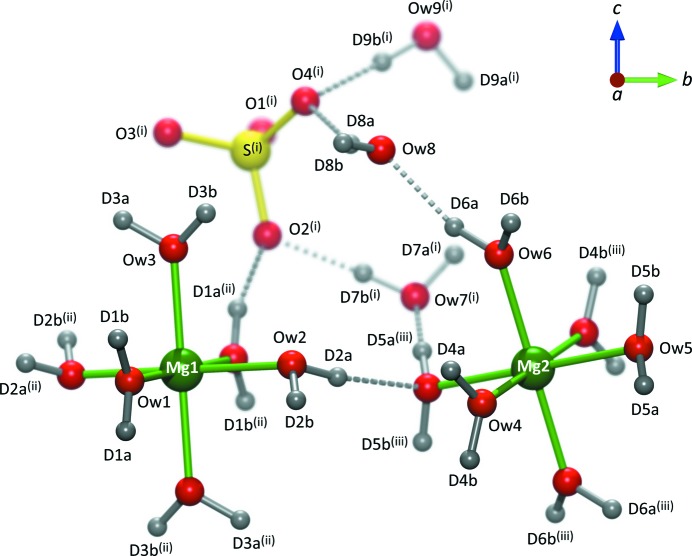
Asymmetric unit of MS9 with some symmetry-related atoms included for clarity. Symmetry codes: (i) 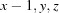; (ii) 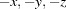; (iii) 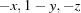. Drawn using *Diamond* (Putz & Brandenburg, 2006 ▸) and rendered with *PovRay* (POV, 2004 ▸).

**Figure 3:**
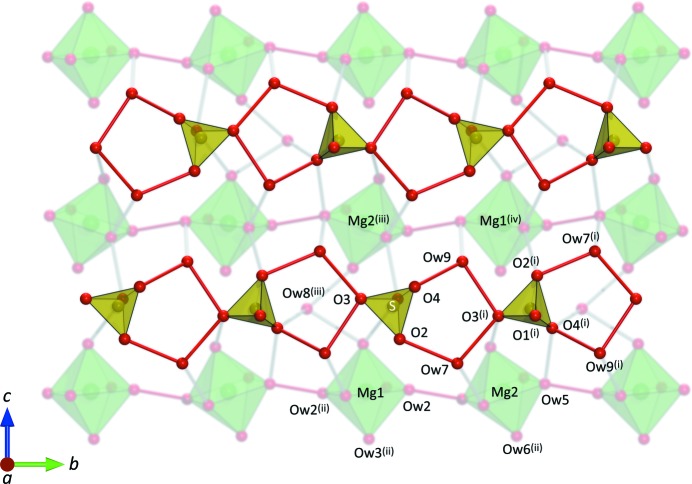
Packing and hydrogen-bond connectivity of the polyhedra in MS9 in the *bc* plane. Symmetry codes: (i) 
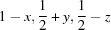
; (ii) 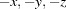; (iii) 
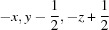
; (iv) 
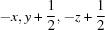
; (v) 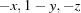. Drawn using *Diamond* (Putz & Brandenburg, 2006 ▸) and rendered with *PovRay* (POV, 2004 ▸).

The structure (Fig. 2 ▸) consists of isolated Mg(H_2_O)_6_ octahedra on sites of 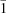 symmetry; the Mg1 octahedron, which occupies the 2*a* position, donates a hydrogen bond (*via* water molecule O*w*2) to its neighbouring octahedron, situated on the 2*c* site, *via* water molecule O*w*5. This effectively creates hydrogen-bonded chains of Mg(H_2_O)_6_ octahedra running parallel to the *b*-axis at *a* = 0. Sulfate tetrahedra sit at *a* ≃ 0.5 linked by the interstitial water molecules O*w*7 and O*w*9 in a pentagonal motif to form ribbons, which also run parallel to the *b*-axis (Fig. 3 ▸). The third interstitial water molecule, O*w*8, cross-links adjacent chains of MgO_6_ octahedra along the *c*-axis, accepting hydrogen bonds from O*w*3 (Mg1-coordinated) and O*w*6 (Mg2-coordinated).

The sulfate O atoms O1, O3 and O4 each accept three hydrogen bonds, whilst O2 accepts only two. The effect of this is typically manifested in the S—O bond lengths, those with the least number of hydrogen-bond connections tending to have the shortest S—O length. Whilst this is evident from the DFT calculations (Table 1 ▸), the differences in S—O bond lengths obtained experimentally show no such systematic variation.

Both Mg(H_2_O)_6_ octahedra are elongated along the axis of the inter-octahedral hydrogen-bond donor–acceptor (Mg1—O*w*2 and Mg2—O*w*5). The occurrence of hydrogen-bond donation to cation-coordinated water is fairly common in divalent metal sulfate hydrates, although it does not occur in the non-isotypic crystal MgSeO_4_·9H_2_O (Fortes *et al.*, 2015 ▸). The 2*c* octahedral sites are larger and more regular (in terms of bond-angle variance) than the 2*a* sites in both Mg and Ni endmembers.

In the majority of inorganic hydrates the cation is either fully saturated (*i.e.* its first coordination shell is entirely filled by water O atoms) or is undersaturated, such that some of the cation-coordinated O atoms are O^2−^ ions rather than neutral H_2_O; this tends to result in corner- and/or edge-sharing polyhedra. In ‘cation-saturated’ hydrates, the water molecules generally donate hydrogen bonds to the anions (or oxyanions) and less frequently donate hydrogen bonds to other water molecules, thus the role of water is to act as a bridge between the ions rather than associating with any neighbouring neutral water.

In hydrates where there is excess water above that required to saturate the cation, the question then arises as to whether the additional water just serves to extend these bridges or whether there is an opportunity for association with neighbouring water to form some kind of polymeric unit (*e.g.* clusters, ribbons or sheets). The structures of both MgSeO_4_·9H_2_O and MS11 contain quite large clusters of hydrogen-bonded water; the selenate 9-hydrate contains a centrosymmetric dodecamer and the sulfate 11-hydrate contains a centrosymmetric hexadecamer. The structure of MgSO_4_·9H_2_O, on the other hand, has no such extended polymeric water structure. What we see instead are a trimer and a linear hexamer (Fig. S4). The trimers are very common in highly hydrated materials such as these, and identical polymeric units are found in MS11, MgSeO_4_·9H_2_O (Fortes *et al.*, 2015 ▸) and Fe^3+^(ClO_4_)_3_·9H_2_O (Hennings *et al.*, 2014*a* ▸), for example.

Since we expect the local structure of the aqueous solution from which these compounds are obtained to be rich in five- and six-sided rings, the occurrence of closed ring-like structures in MS11 and MgSeO_4_·9H_2_O is unsurprising. However, it appears that these are substantially less common than finite branched and unbranched chains. Branched or decorated (H_2_O)_9_ chains occur in both trivalent and divalent metal bromide and iodide enneahydrates (Hennings *et al.*, 2013 ▸; Schmidt *et al.*, 2014 ▸), whereas branched (H_2_O)_8_ chains appear in CaBr_2_.9H_2_O (Hennings *et al.*, 2014*b* ▸). Of course, the principal difference with our title compound is the 1:2 or 1:3 cation-to-anion ratio in these other materials. We might anticipate that in the 1:1 cation-to-anion compounds with increasing numbers of water molecules per formula unit that the interstitial water would be free to adopt a more liquid-like arrangement, comprising closed rings. That it does not do so in MS9, forming instead a linear six-membered chain, may be related to its metastability.

### 3.2. DFT structural relaxations   

The structure of MS9 was optimized using both the PBE and WC functionals starting from the 9 K experimental structure. The unit-cell parameters and atomic coordinates output from these simulations are provided in the supporting CIFs. We found that PBE led to an athermal unit-cell volume ∼ 4.5% larger than the experimental value measured at 9 K, whereas the WC functional gave a zero-pressure athermal cell just 1% smaller (Table 1 ▸). Both S—O and Mg—O bond lengths are in closer agreement with experiment in the WC GGA calculations, but are still over-estimated. In the case of both functionals, the correlation between observed and calculated O—H⋯O angles and H⋯O distances is very good, which is to say that the overall intermolecular connectivity is well reproduced. PBE and WC yield closely similar hydrogen-bond angles but differing hydrogen-bond lengths; PBE produces hydrogen bonds that are too weak and WC produces hydrogen bonds that are slightly too strong. The difference in the calculated molar volumes between the two GGA functionals therefore arises principally from an average decrease of 4 ± 2% in the length of H⋯O hydrogen bonds.

The calculations capture differences in S—O bond lengths due to differences in the number of hydrogen bonds accepted by the apical O atoms (notably, S—O2), a feature that is not seen clearly in the experimental powder refinement. This is indicative of a level of ‘noise’ in the structural parameters arising from the multi-phase powder refinement, since these subtle differences are typically seen very clearly in single-crystal data from other similar materials (*e.g.* Fortes *et al.*, 2015 ▸). The shape of the M2 octahedron is well reproduced, being elongated along the Mg2—O*w*5 axis as a result of the hydrogen bond accepted by O*w*5. However, both the PBE and WC calculations reveal a similar distortion of the M1 octahedron, this being elongated along the Mg1—O*w*3 axis, for which there is no obvious rationale since O*w*3 is quite unambiguously in trigonal coordination, *i.e.* it does not accept a hydrogen bond. In this instance the neutron powder refinement shows the ‘expected’ pattern of bond lengths for the M1 octahedron.

Since the overall structural parameters produced by the WC GGA calculations are in such excellent agreement with the observations a choice was made to proceed with further work using only the WC functional.

**Figure 4:**
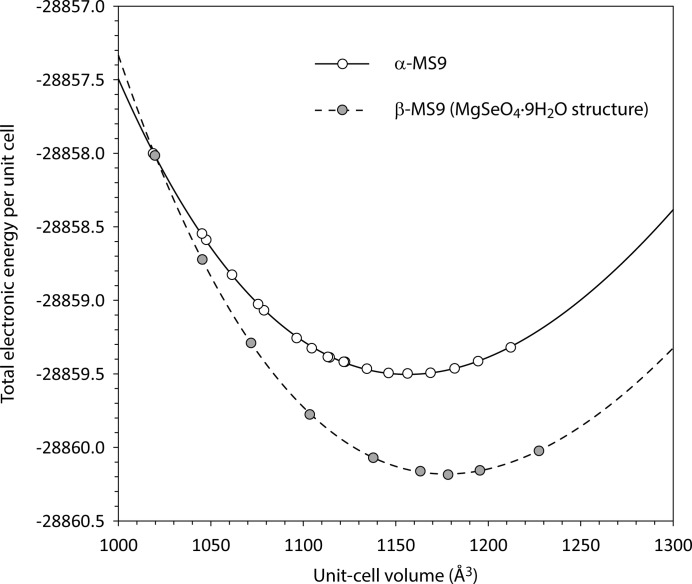
Calculated variation of total electronic energy against unit-cell volume for the experimentally observed structure of MgSO_4_·9H_2_O (α-MS9) and a hypothetical polymorph with the same structure as MgSeO_4_·9H_2_O, dubbed β-MS9. Solid and dashed lines are integrated third-order Birch–Murnaghan equations of state fitted to the calculation results (open and filled symbols). For α-MS9 the fit parameters are *V*_0_ = 1156.4 (5) Å^3^, *K*_0_ = 24.2 (6) GPa and *K*′_0_ = 3.7 (5). For β-MS9 the equivalent fit parameters are *V*_0_ = 1177.1 (4) Å^3^, *K*_0_ = 25.6 (5) GPa and *K*′_0_ = 4.4 (3).

Geometry optimizations were carried out on MS9 at a range of fixed pressures from −2 to + 5 GPa. Fig. 4 ▸ shows a plot of the total energy of the crystal as a function of unit-cell volume in this pressure range; zero-pressure corresponds to the minimum in the curve, where the gradient is zero, since *P* = −d*E*/d*V*.

We also evaluated the stability of a possible sulfate analogue to MgSeO_4_·9H_2_O. The selenate is stable in aqueous solutions at low temperature, allowing growth of large single crystals, but it is not isotypic with the sulfate described in this paper. However, it is trivial to carry out geometry optimizations on this structure with sulfur substituted for selenium. As shown in Fig. 4 ▸, this hypothetical polymorph (termed β–MS9) is marginally less dense than the observed polymorph (α-MS9), having a molar volume at zero pressure (the minimum in the dashed curve) that is ∼ 1.8% larger, and it has a lower total electronic energy at volumes greater than 1020 Å^3^ per unit cell. More specifically what this means is that the α-MS9 structure is not the thermodynamically stable form of MgSO_4_·9H_2_O at ambient pressure; there is an alternative structural arrangement with the same composition (β-MS9) having a lower enthalpy at 0 K. The enthalpy difference between the two phases at zero pressure amounts to 17 (1) kJ mol^−1^ (in favour of β-MS9) and the point at which the enthalpy difference, Δ*H* = 0, occurs is at 4.3 (4) GPa. In §3.4 we will also show that α-MS9 is unstable with respect to both MgSO_4_·11H_2_O and MgSO_4_·7H_2_O, depending on the temperature and availability of water vapour, and we must also assume that there is a sufficient kinetic barrier even at 250 K to prevent the transformation of α-MS9 into the hypothetical β-phase. A CIF containing the zero-pressure structural parameters of β-MS9 is provided in the supporting information.

### 3.3. Thermal expansion   

**Figure 5:**
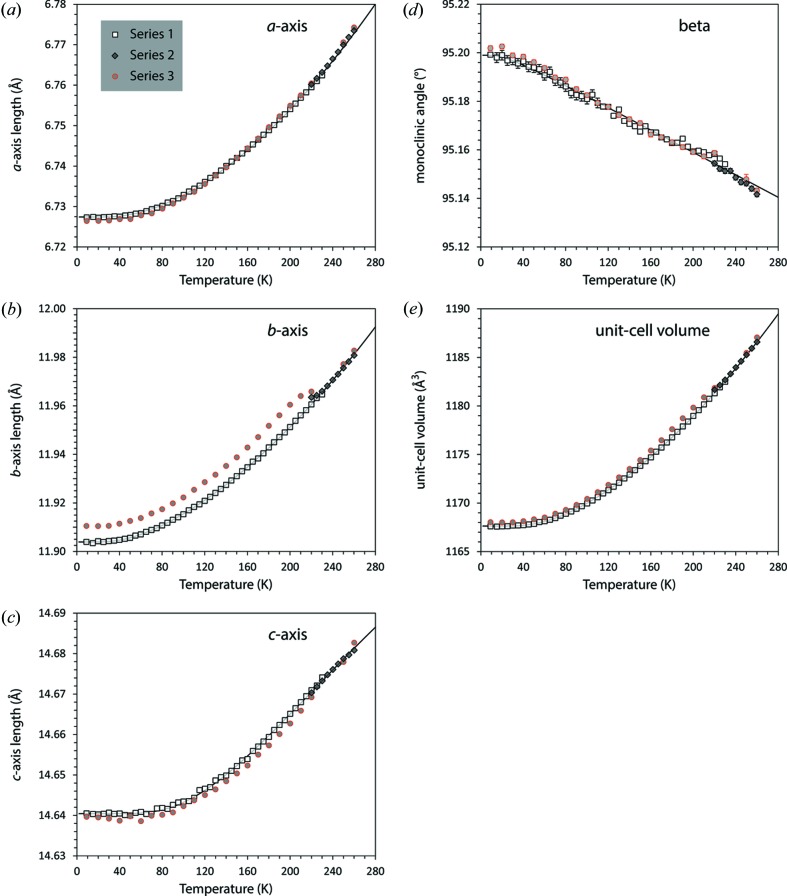
Unit-cell parameters of MS9 as a function of temperature; solid lines represent unweighted fits of a modified Einstein expression [equations (1), (2) and (3)] to the Series 1 data, done using *Origin Pro* (OriginLab, Northampton, MA).

Lattice parameters were refined at 45 temperatures from 9 to 230 K, in 5 K increments (Series 1), nine temperatures from 220 to 260 K in 5 K increments (Series 2), and 24 temperatures from 9 to 260 K in 10 K increments (Series 3). These values are given in Table S1 and plotted in Fig. 5 ▸.

It is quite obvious that not all of the cell-parameter data are continuous. This is most clear in the *b*-axis data, although both *a* and *c* exhibit more subtle signatures, where a relaxation process occurs on warming through ∼ 220 K. In Series 1, this effect appears to be absent, with all parameters forming smooth curves that are, importantly, contiguous with the higher-temperature portions of Series 2 and 3. We infer that the Series 1 cell parameters represent the most completely annealed strain-free state of the crystal at all temperatures. Series 3 has a significantly different *b*-axis length to the other observations, which ‘turns over’ at 220 K to join Series 1 and 2. Series 2 exhibits a similar small ‘kink’ at the lowest temperature; taken together these seem to show a structural relaxation along the unique axis of the crystal into a more stable state. The temperature at which this structural relaxation occurs is in the same region where the progenitor glass devitrifies. The origin of the phenomenon is not clear, since we have no high-precision structural datasets in this region and there are no orientationally disordered water molecules in the structure. One possible explanation is that Bjerrum defects (such as two protons occupying the O*w*2⋯O*w*5 bond) could lead to expansion along *b*, although we have no data with which to test this hypothesis.

For the purpose of making a simple density calculation (*e.g.* for planetary modelling) we have fitted a second-order polynomial to the calculated density of MS9 between 80 and 260 K, ρ = *AT* ^2^ + *BT* + ρ_0_ assuming that the molar volumes of the protonated and deuterated species are identical. The coefficients obtained are *A* = −3.25 (5) × 10^−7^ g cm^−3^ K^−2^, *B* = −2.4 (2) × 10^−5^ g cm^−3^ K^−1^ and ρ_0_ = 1.6095 (1) g cm^−3^ for protonated MS9, valid only between 80 and 260 K.

In order to interpret the subtleties of the thermal expansion tensor, we analyse the data in terms of an Einstein oscillator model similar to that used previously for analysis of the thermal expansion of MS11 (Fortes *et al.*, 2008 ▸) where all atoms in the solid vibrate at the same characteristic angular frequency, ω_E_, which is expressed in terms of an Einstein temperature, θ_E_ = 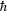
ω_E_/*k*_B_. The advantage of this with respect to the more complex double-Debye model required to fit these data accurately is that fewer adjustable parameters are needed, as shown below.

The temperature dependence of the molar volume, *V*(*T*) is described as
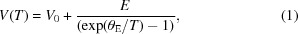
where *E* = 3*R*γθ_E_/*K*_T_ (γ is the Grüneisen ratio, *K*_T_ is the isothermal bulk modulus and *V*_0_ is the zero-temperature molar volume). Note that this is only dimensionally correct when *V* is a volume in m^3^ mol^−1^.

A similar expression may be used to describe the temperature dependence of the lattice parameters
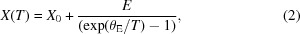
where *X*_0_ is the value of the fitted parameter at 0 K. For the purpose of deriving the coefficients of the thermal expansion tensor a sufficiently good fit is only obtained when the parameter *E* is allowed to vary linearly as a function of temperature
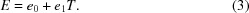
The parameters obtained from fitting equation (1) [and (3)] to the unit-cell volume of MS9, and fitting equation (2) [and (3)] to the individual cell parameters, are listed in Table 3 ▸ and the fits are depicted in Fig. 5 ▸ as solid lines.

This formalism is not as accurate as the more rigorous Debye model, which would be the preferred choice when heat capacity data are available to aid in constraining the Debye temperatures. Nevertheless, we present the fitting of two different Debye models in the supporting information.

Since for a crystal of monoclinic symmetry, two of the three principal directions of the thermal expansion tensor are free, whilst remaining orthogonal to one another, to rotate freely about the crystal’s unique axis, an accurate picture of the structure’s behaviour is not readily obtained by only fitting expressions to the individual axial lengths as a function of temperature. We therefore use the Einstein model fits to the cell parameters in order to derive the thermal expansion tensor coefficients. For a monoclinic crystal these are represented by a symmetrical second rank tensor of the form
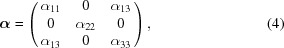
the components of which are unit strains corresponding to thermal expansion coefficients. The eigenvalues and eigenvectors of this matrix, obtained by matrix decomposition methods, are the magnitudes and orientations of the three principal axes of the thermal expansion tensor (*i.e.* directional expansivities, 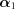, 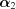 and 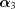) with respect to an orthogonal basis. We have applied the commonly used Institute of Radio Engineers relationship between the orthogonal basis and the unit-cell of a monoclinic crystal such that *X* || a*, *Y* || *b* and *Z* || *c* (Boisen & Gibbs, 1990 ▸).

The fitted parameters in Table 3 ▸ were used to calculate smoothly varying unit-cell parameters as a function of pressure, from which the thermal expansion coefficients were obtained using the methods described by Schlenker *et al.* (1978 ▸) and Hazen & Finger (1982 ▸); the magnitudes of the principal expansivities, 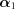, 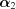 and 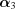, and their spatial orientation with respect to the Cartesian basis were then obtained by standard eigenvalue decomposition methods.

**Figure 6:**
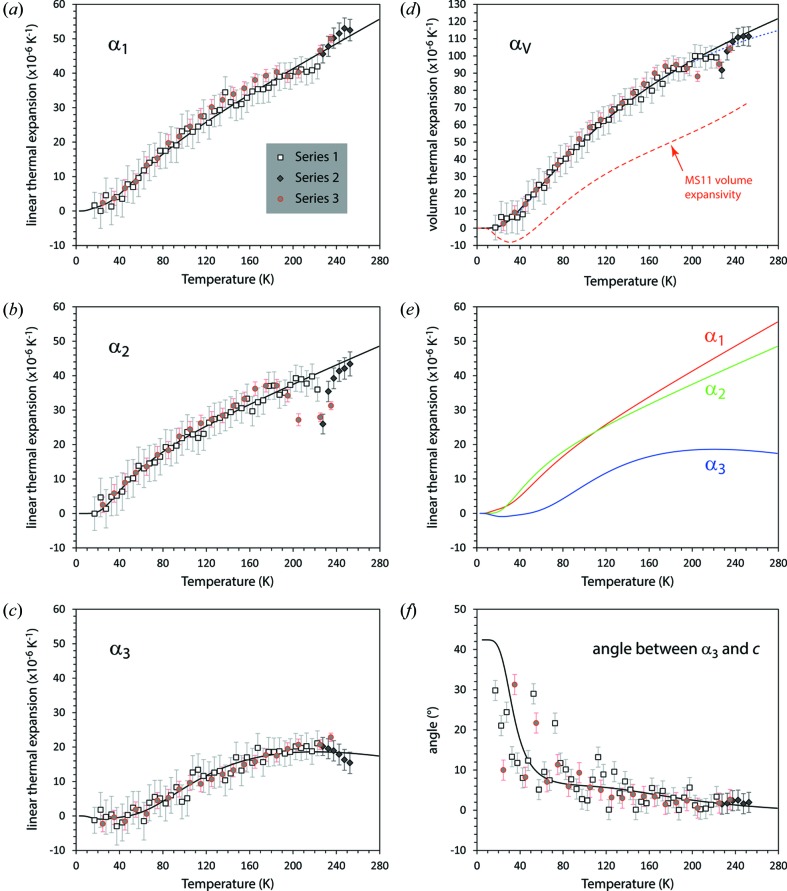
Principal and volume thermal expansion coefficients of MS9 as a function of temperature. Solid lines are derived from smoothed values calculated using the Einstein-model fit parameters reported in Table 3 ▸. Symbols are ‘point-by-point’ derivatives of the raw cell parameter values calculated across a moving window 15 K wide. The dashed red line in (*d*) depicts the volume thermal expansion of MS11 for comparison with that of MS9: the dotted blue line represents the unconstrained double-Debye model fit to the data (see text in the supporting information)

The principal and volumetric thermal expansivities are shown in Fig. 6 ▸; symbols with error bars depict point-by-point derivatives of the ‘raw’ unit-cell parameters calculated (in order to reduce the uncertainties) across moving 15 K wide windows.

**Figure 7:**
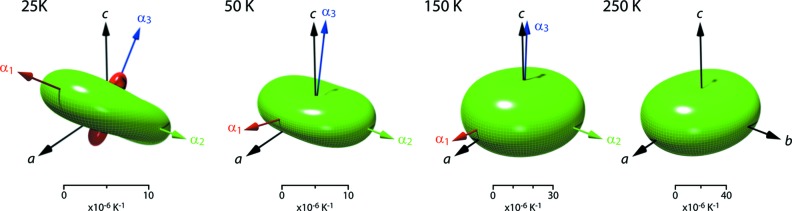
Representation surface of the MS9 thermal expansion tensor at selected temperatures. Coloured arrows identify the eigenvectors of the thermal expansion tensor and the black arrows show the crystallographic axes. Green shading indicates positive values and red shading indicates negative thermal expansion values. Tensor surfaces were generated in *WinTensor* (Kaminsky, 2004 ▸ 2007 ▸) and postprocessed with *Meshconv* (courtesy of Patrick Min, Princeton) and *Meshlab* (Sourceforge).

Representation surfaces (Reynolds glyphs – see Hashash *et al.*, 2003 ▸) of the unit-strain tensor – or more specifically in this case, the thermal expansion tensor – at a range of temperatures are shown in Fig. 7 ▸, where the distance from the origin to the edge of the representation surface indicates the thermal expansivity in any given direction.

The absolute values of the volume thermal expansion coefficient are large when compared with other similar cryohydrates. The temperature dependence of the volume expansivity of MS11(D) is drawn in Fig. 6 ▸(*d*); for comparison the volume expansivity of MS11(D) at 250 K is ∼ 72 × 10^−6^ K^−1^, that of MS7(D) is ∼ 89 × 10^−6^ K^−1^ (Fortes *et al.*, 2006 ▸) and that of Na_2_SO_4_·10D_2_O is ∼ 90 × 10^−6^ K^−1^ (Brand *et al.*, 2009 ▸). However, the volume thermal expansivity of MS9 is virtually identical to the non-isostructural MgSeO_4_·9D_2_O, these being 113 and 110 × 10^−6^ K^−1^, respectively, at 250 K and the two materials exhibit large degrees of anisotropy (Fortes, 2016 ▸). For MS9(D), at 250 K, the eigenvalues of the thermal expansion tensor are fairly isotropic in the *a*–*b* plane (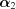/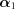 = 0.88) whilst the eigenvalue most closely aligned with *c* is substantially smaller (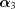/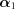 = 0.35). In MgSeO_4_·9D_2_O at 250 K the direction of least thermal expansion is aligned with the twofold axis, which we define as 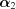; hence, 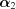/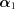
= 0.09 and 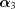/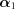 = 0.63. It is also noteworthy that both sulfate and selenate 9-hydrates lack the region of negative volume expansion observed in MgSO_4_·11D_2_O and Na_2_SO_4_·10D_2_O.

In MS9 the angle between 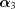 and *c* is negligibly small at high temperatures and only increases to > 5° below 100 K. A fairly dramatic tilt of the representation figure below 50 K corresponds to 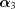 turning negative and the development of a cone of pure shear between the positive and negative lobes of the strain figure.

### 3.4. Phase transitions at constant *T* and on warming   

In the vast majority of MS9 syntheses, water ice is present in substantial quantities. We observed, however, that the intensity of ice peaks would diminish during the acquisition of X-ray powder diffraction data at *ca.* 250 K at the same time as which additional Bragg peaks from MS11 would appear. For measurements of many hours duration, this would continue until virtually all detectable ice in the specimen had been consumed. Our interpretation of this was that MS9 reacts with water vapour in pore spaces and at the specimen’s large air interface, to form the thermodynamically stable phase, MS11. Since the equilibrium vapour pressure of ice is quite substantial, approaching 1 mbar at the temperature of the X-ray measurements (92.4 Pa at 252 K: IAPWS, 2011 ▸; Wagner *et al.*, 2011 ▸), the vapour consumed by reaction with MS9 must be replaced by further sublimation of ice. Consequently, the rate at which this reaction proceeds, being a function of the vapour pressure of ice, will be strongly dependent on temperature. Moreover, at a given temperature, it should be slower for deuterated specimens than protonated samples by virtue of the low vapour pressure over D_2_O ice (*P*_vap_[D_2_O] = 67 Pa at 252 K: Matsuo *et al.*, 1964 ▸).

A series of X-ray powder diffraction patterns were obtained from a sample containing MS9 + ice with negligible initial quantities of MS11 over a period of several hours, measuring in the 2θ range 5–50° every 20 min (Fig. S5); clearly, peaks from MS11 grow over time at the expense of both MS9 and water ice.

**Figure 8:**
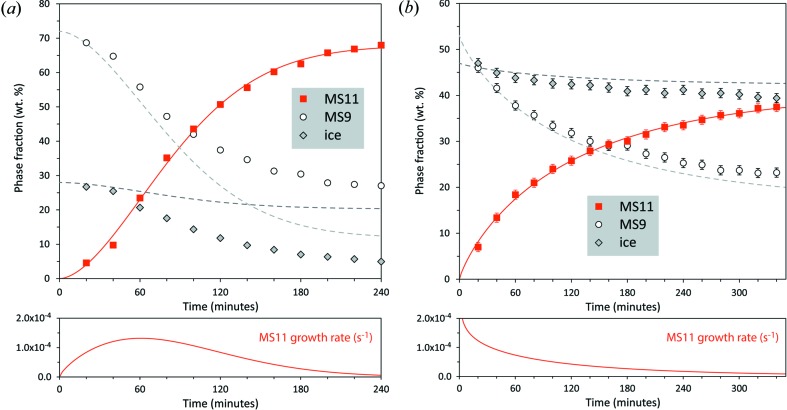
(*a*) Refined phase fractions of MS9, MS11 and ice as a function of time from X-ray powder diffraction data measured on a Peltier-cooled cold stage with *ca* 52 cm^3^ head-space over the sample. The solid line through the symbols for MS11 reports the fit of equation (5) to the data. The two dashed lines show the expected phase fractions of ice and MS9 concomitant with the observed abundance of MS11 as a function of time. (*b*) Refined phase fractions of deuterated MS9, MS11 and ice as a function of time from neutron powder diffraction data measured in an indium-sealed container. Lines and symbols have the same meaning as in part (*a*).

These XRD data were used to refine the phase fractions of each constituent as a function of time (Fig. 8 ▸ *b*). The well known Kolmogorov–Johnson–Mehl–Avrami (KJMA) expression (Kolmogorov, 1937 ▸; Johnson & Mehl, 1939 ▸; Avrami, 1939 ▸, 1940 ▸) was fitted to these data
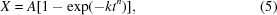
where *X* is the MS11 phase fraction, *A* is the saturation value of the phase fraction, *k* is a rate constant (units time^−*n*^), and *n* is the ‘Avrami exponent’, which contains information on the time dependence of nucleation, the geometry of the growing crystallites and the nature of the reaction process (interfacial *versus* diffusional). The Avrami exponent *n* is the sum of two quantities, α + β. The number density of nucleation centres (*N*) has a model time dependence of the form *N* ∝ *t* ^α^, such that for α = 0 all nucleation sites are present at *t* = 0 (the growth medium is nuclei site saturated), and for α = 1 the nucleation rate is a constant: for α < 1 the nucleation rate slows with time, and for α > 1, the nucleation rate increases with time. The term β expresses the dimensionality of the growth geometry (*i.e.* one-dimensional = needle-like or acicular growth, two-dimensional = platy or tabular growth, three-dimensional = blocky or globular growth), and has the values 1, 2 or 3 for one-dimensional, two-dimensional or three-dimensional growth, respectively, when the reaction process is interfacial, and has the values 0.5, 1 or 1.5 when the reaction process is diffusional. For our purposes, we can infer to a certain extent the nature of the reaction process, and also use the known habit of MS11 to infer that the growing crystallites will be platy.

The data in Fig. 8 ▸(*a*) are fitted with equation (5) to obtain *A* = 0.68 (1), *k* = 2.9 (4) × 10^−7^ s^−*n*^ and *n* = 1.73 (8). Thus, from the X-ray powder data collected at 252 K, we find that the peak growth rate is 1.3 × 10^−4^ s^−1^, which occurs after 1.0 h. It is clear from Fig. 8 ▸(*a*) that the consumption of MS9 is smaller, and that of ice larger, than is commensurate with the observed abundance of MS11. This suggests that, in the thin surface layer of the sample being probed by X-rays, water ice is being preferentially depleted by another process – most probably sublimation: in other words, the observed phase mixture is not determined *only* by the reaction MgSO_4_·9H_2_O + 2H_2_O → MgSO_4_·11H_2_O.

In fact we have the possibility to test this hypothesis by observing the same process in a confined bulk sample using a neutron diffraction probe (Series 3). Fig. S6 shows the growth of MS11 Bragg peaks at the expense of MS9 and ice at 260 K over a period of 5.5 h in an indium-sealed can. As before, the refined phase fraction of MS11 has been fitted with equation (5) to obtain *A* = 0.41 (1), *k* = 6.0 (2) × 10^−4^ s^−*n*^ and *n* = 0.83 (4). As shown in Fig. 8 ▸(*b*), we now find that the expected abundances of both MS9 and ice are in closer agreement with the observations, reflecting the limited pore space and head space in the sample can that is available for saturation with vapour from sublimated water ice.

Evidently, the peak growth rate has passed at the onset of the 260 K neutron measurements, and the rate we observe declines as a function of time. This suggests that growth began (and peaked) during the phase of warming from 250 to 260 K and/or the subsequent short period of thermal equilibration. In terms of the Avrami exponent, the most logical interpretation is that growth of MS11 is two-dimensional (platy) and diffusional, giving β = 1. The difference between the two observations then is that nucleation occurs slowly over the course of the X-ray measurement at 252 K (*i.e.* α ≃ 1), whereas the slightly higher temperature in the neutron measurement [and associated increase in vapour pressure, *P*_vap_(D_2_O) = 147 Pa] results in rapid saturation of nucleation centres (*i.e.* α ≃ zero). This produces *n* ≃ 2 for the X-ray measurements and *n* ≃ 1 for the neutron measurements, close to what we obtain and consistent with both the expected morphology and crystal growth process.

In light of the transformation of MS9 to MS11 in the presence of ice, or more accurately water vapour in equilibrium with ice, we made considerable efforts to synthesize ice-free samples. As described in §2.1, we were able to achieve this by careful evaporation of MgSO_4_ solutions into a supersaturated state at ∼ 383 K followed by flash freezing in liquid nitrogen. Fig. S7 shows X-ray powder diffraction patterns obtained from just such an ice-free specimen both before and after a 12 h data collection. The objective was to form an initial liquid containing 42.6 wt % MgSO_4_, which would freeze to form phase-pure MS9. The XRD data reveal a small amount of MS11 is present with a refined phase fraction of 4.8 (1) wt %; this corresponds to a bulk composition for the sample of 42.4 wt % MgSO_4_ – very close indeed to our target.

After 12 h, the accessory MS11 has disappeared and we see instead Bragg peaks from MS7, epsomite, with a refined phase fraction of 15.7 (1) wt %. The bulk composition of the sample is now 43.6 wt % MgSO_4_, indicating a small net loss of water. The observation requires all of the initial MS11 and ∼ 11% of the MS9 to have dehydrated to MS7 in the cold, dry conditions of the sample holder with the loss (depending on initial packing density) of between 2 and 4 mg of water. This is sufficient, if confined solely to the sample pore space, to produce a partial pressure of water vapour of some tens of Pascals. It would be useful to characterize this process in more detail, both as a function of temperature and relative humidity so as to place this process in context with what we know about the transformation of other MgSO_4_ hydrates under similar conditions (Chou & Seal, 2004 ▸; Chipera & Vaniman, 2007 ▸; Wang *et al.*, 2009 ▸, 2011 ▸). Such information is essential to determining the longevity of MS9 as a mineral in, for example, the martian regolith.

### 3.5. Isothermal equation of state   

Data Series 4, measured in a TiZr pressure vessel under He gas, yielded unit-cell parameters for MS9(D) at six state points between 10 and 543 MPa at 240 K. One additional datum at 1.07 (2) GPa was obtained by refinement of a neutron powder pattern from a mixture of MS9(D) and deuterated ice VI formed by the dissociation of MS11(D) (see Fortes *et al.*, 2017 ▸). Cell parameters as a function of pressure are given in Table S2.

A convenient description of the material’s stiffness may be obtained by parameterizing the pressure-dependence of the unit-cell volume using a Murnaghan integrated linear equation of state, MILEOS (Murnaghan, 1944 ▸)
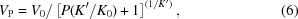
where *K*_0_ is the zero-pressure isothermal bulk modulus and *K*′ is the first pressure derivative of the bulk modulus, (∂*K*_0_/∂*P*). An unweighted fit of equation (6), done using *OriginPro*, to the experimental *V*(*P*) obtained solely from the gas cell study on HRPD yields *V*_0_ = 1184.9 (2) Å^3^, *K*_0_ = 19.3 (6) GPa and *K*′ = 4 (2): inclusion of the data point from the higher-pressure study on PEARL/HiPr improves the precision most noticeably on the value of *K*′, with *V*_0_ = 1184.8 (2) Å^3^, *K*_0_ = 19.5 (3) GPa and *K*′ = 3.8 (4). Hence the incompressibility of MS9(D) is virtually identical to synthetic meridianiite (*K*_0_ = 19.9 GPa; Fortes *et al.*, 2017 ▸) and synthetic mirabilite (K_0_ = 19.1 GPa: Brand *et al.*, 2010 ▸) and MS9(D) is somewhat more compressible than synthetic epsomite (K_0_ = 22.6 GPa: Fortes *et al.*, 2006 ▸).

As we have done for the thermal expansion, the elastic strain due to a hydrostatic stress is described by a symmetrical second rank tensor of the form
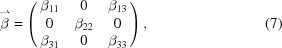
the components of which are unit strains corresponding to compressibility coefficients. The eigenvalues and eigenvectors of this matrix, are the magnitudes and orientations of the three principal axes of the elastic strain figure (*i.e.* directional compressibilities, 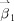, 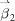 and 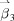) with respect to the same orthogonal basis as was used in the derivation of the thermal expansion tensor.

**Figure 9:**
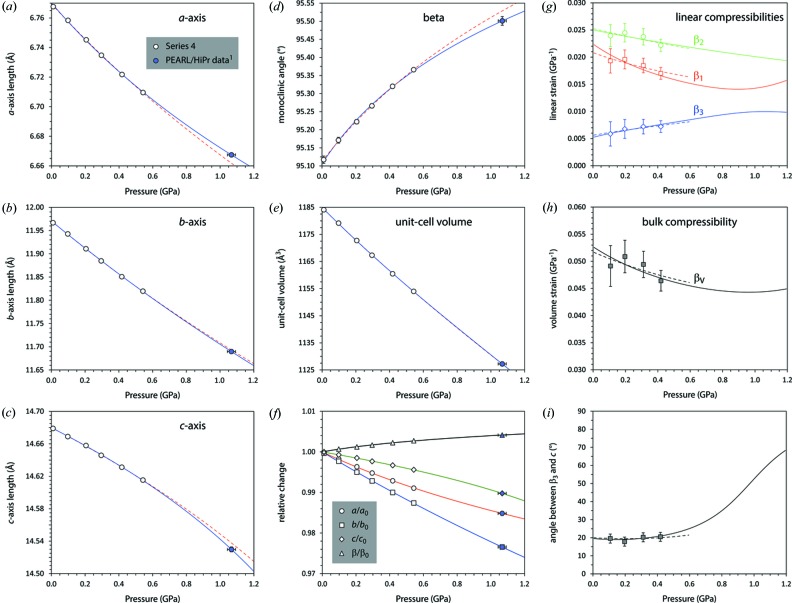
Unit-cell parameters of MS9 as a function of pressure at 240 K. Open symbols correspond to data measured in a TiZr gas cell on HRPD (Series 4), whereas the filled symbol was measured in a Paris–Edinburgh press on PEARL/HiPr (Fortes *et al.*, 2017 ▸). Solid lines are least-squares fits of equation (6) to all of the data, whereas the dashed red lines are fits only to the gas cell data. The fitted parameters are listed in Table 4 ▸. Panel (*f*) summarizes the relative variation of each unit-cell parameter with pressure. (*g*) Principal linear compressibilities and (*h*) volume compressibility of MS9 at 240 K. Solid lines are derived from fitting equation (6) to both the HRPD and PEARL/HiPr data, whereas the dashed lines are derived from fitting only to the HRPD gas-cell data. Symbols with error bars show the moving-average derivatives calculated from the observed unit-cell parameters. Panel (*i*) shows the angle between the stiffest direction in the crystal, 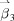 and the *c*-axis.

Each of the unit-cell parameters was fitted with a Murnaghan integrated linear equation of state of the same form as equation (6): the parameters resulting from these fits are listed in Table 4 ▸. A plot of the high-pressure unit-cell parameters is given in Fig. 9 ▸ with solid and dashed lines reporting various MILEOS fits to each.

The fitted parameters in Table 4 ▸ were used to calculate smoothly varying unit-cell parameters as a function of pressure, from which the Eulerian infinitesimal unit-strain coefficients were obtained using the method outlined in §3.3; the magnitudes of the principal compressibilities, 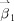, 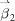 and 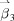, and their spatial orientation with respect to the orthogonal basis were then obtained by standard eigenvalue decomposition methods. Fig. 9 ▸(*g*) shows the linear compressibilities derived from the raw experimental data and from the MILEOS fits where, as in the rest of Fig. 9 ▸, dashed lines correspond to fits only to the gas cell data and solid lines include the PEARL/HiPr point at 1.07 (2) GPa.

The principal and volumetric compressibilities and incompressibilities (*e.g.* 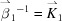) were fitted with linear functions in *P* (in the low-pressure limit) to obtain the zero-pressure values and their first derivatives (Table 5 ▸). It is worth observing that this method recovers with reasonable accuracy the values of *K*_0_ and *K*′ found earlier by fitting the Murnaghan integrated linear equation of state. It is apparent from Fig. 9 ▸(*g*) that the stiffest principal direction, 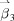, softens under compression – indeed this can be seen by visual inspection of the *c*-axis as a function of pressure (Fig. 9 ▸ *c*). What Fig. 9 ▸(*g*) shows is that the softening of 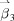 is reduced above ∼ 1 GPa whilst the slope of 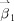 (which had hitherto been stiffening at a high rate) turns positive. The overall effect of this is to cause the *bulk* compressibility of the crystal to increase above ∼ 1 GPa (Fig. 9 ▸ *h*). This change in the eigenvalues of the strain tensor is matched by a large change in the eigenvectors; Fig. 9 ▸(*i*) shows that the angle between 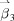 and the *c*-axis increases by ∼ 50° from 0 to 1.2 GPa.

**Figure 10:**
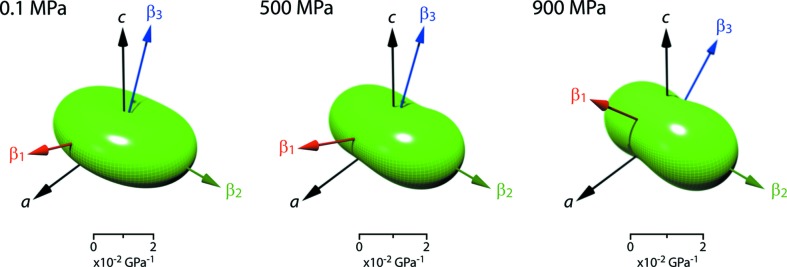
Representation surface of the MS9 unit-strain figure at selected pressures. Coloured arrows identify the eigenvectors of the compressibility tensor and the black arrows show the crystallographic axes. Tensor surfaces generated in *WinTensor* (Kaminsky, 2004 ▸, 2007 ▸) and post-processed with *Meshconv* (courtesy of Patrick Min, Princeton) and *Meshlab* (Sourceforge).

Representation surfaces of the elastic strain figures at zero pressure, 500 and 900 MPa are shown in Fig. 10 ▸ where the distance from the origin to the edge of the representation surface indicates the compressibility in any given direction under hydrostatic stress.

There are some noteworthy similarities between the strain response of the crystal to both thermal and hydrostatic stress. The crystal in both instances exhibits the largest fairly isotropic strains in a plane that is roughly perpendicular to *c* whilst exhibiting the smallest strain roughly parallel with *c*.

### 3.6. DFT equation of state   

**Figure 11:**
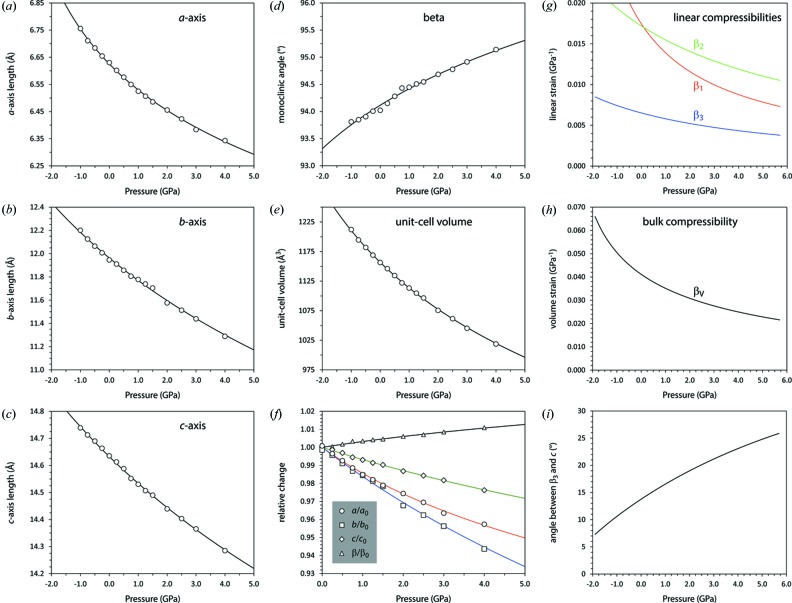
Unit-cell parameters of MS9 as a function of pressure from DFT calculations (athermal, WC GGA). Solid lines are least-squares fits of equation (6), the fitted parameters being listed in Table 4 ▸. Panel (*f*) summarizes the relative variation of each unit-cell parameter with pressure. (*g*) Principal linear compressibilities and (*h*) volume compressibility of MS9 from DFT calculations (athermal, WC GGA). Panel (i) shows the angle between the stiffest direction in the crystal, 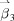, and the *c*-axis.

The energy–volume curves (Fig. 4 ▸) were each fitted with an integrated form of the third-order Birch–Murnaghan equation of state in order to determine the zero-pressure athermal molar volume, the zero-*P*/*T* bulk modulus and the first pressure derivative of the bulk modulus. The parameters from these fits are given in the caption to Fig. 4 ▸. Since our calculations also provide us with relaxed cell-parameters at each pressure point (Figs. 11 ▸ *a*–*d*) we are able to reproduce the derivation given in §3.5 of the elastic strain tensor coefficients. The pressure dependence of the principal axial compressibilities and the volume compressibility are shown in Figs. 11 ▸(*g*)–(*i*). Parameters evaluated at zero *P* and *T* are reported in italics in Table 5 ▸ for comparison with the experimental values at 240 K.

The pressure dependences of the unit-cell parameters are in very good agreement with the experimental observations – with one important exception; they do not reproduce the increasing compressibility of the *c*-axis and the associated increase in bulk compressibility. This difference may be due entirely to the influence of temperature. However, obtaining further insight through finite-temperature calculations represents a formidable computational expense. Our experimental observation of the pressure-induced softening seems robust, being present in data both with and without the additional point obtained in the Paris–Edinburgh press. An improved high-pressure experimental study that straddles the two existing datasets and also samples a wide temperature space is essential before an *ab initio* molecular dynamics study can be justified.

## 4. Possible natural occurrences of MgSO_4_·9H_2_O   

Magnesium sulfate is a quite common constituent of water on Earth, although the number of locations where it is sufficiently concentrated to produce MgSO_4_ hydrate (or cryohydrate) minerals is not large, the stability of the higher hydrates being dependent on low ambient temperatures and high humidity. The highest hydrate, MgSO_4_·11H_2_O, occurs naturally as the mineral meridianiite in a variety of glacial and periglacial environments (Sakurai *et al.*, 2009 ▸; Genceli *et al.*, 2009 ▸) and in a limited number of MgSO_4_-rich hypersaline lakes during the winter months; example localities where the mineral has been identified include the Basque Lakes, Clinton Lake and kłlil’x^w^ (aka Spotted Lake), all in British Columbia, Canada (*e.g.* Peterson *et al.*, 2007 ▸; Cannon, 2012 ▸). Whilst MgSO_4_-rich saline waters are comparatively rare on Earth due to the influence of continental weathering, such liquids are expected to be common on other rocky planets where the weathering of basaltic materials dominates (King *et al.*, 2004 ▸). On Mars, abundant magnesium(II) and iron(III) sulfates are known to occur, including minerals such as kieserite and jarosite (*e.g.* Clark *et al.*, 1976 ▸; Toulmin *et al.*, 1977 ▸; Wänke *et al.*, 2001 ▸; Foley *et al.*, 2003 ▸; McSween, 2004 ▸; Chipera & Vaniman, 2007 ▸) and it is hypothesized that meridianiite may occur in a permafrost-like deposit, forming a substantial reservoir of bound water in the near-surface regolith (Feldman, Mellon *et al.*, 2004 ▸; Feldman, Prettyman *et al.*, 2004 ▸; Peterson & Wang, 2006 ▸). Similarly, water–rock interactions during the accretion and differentiation of icy planetary bodies in the outer solar system may have resulted in large brine reservoirs crystallizing substantial quantities of MgSO_4_ and Na_2_SO_4_ hydrates (Kargel, 1991 ▸). These are apparent in near-IR spectra of their surfaces (Orlando *et al.*, 2005 ▸; Dalton, 2007 ▸; Dalton *et al.*, 2005 ▸; Shirley *et al.*, 2010 ▸), although it remains unclear the extent to which some of the hydrated salts on Europa’s surface are due to endogenic *versus* exogenic processes, such as radiolysis of MgCl_2_ combined with sulfur implantation from neighbouring Io (Brown & Hand, 2013 ▸).

Although MS9 is evidently metastable in the presence of liquid water or water vapour at ambient pressures we have determined that it forms above ∼ 800 MPa at 240 K by the decomposition of MS11 into MS9 + ice VI (Fortes *et al.*, 2017 ▸) in much the same fashion as MgSiO_3_ perovskite in the Earth’s mantle forms when spinel-structured Mg_2_SiO_4_ (ringwoodite) experiences a pressure-induced exsolution of one formula unit of its component oxide. Judging from the inferred high-pressure melting curve of MS11 derived in our companion paper, MS9 may well occur at pressures of 600–800 MPa in the region from 240 to 270 K. The implication of this is that MS9 could be present in substantial quantities as a ‘rock-forming’ mineral in icy satellites that are speculated to have MS11-bearing outer layers of sufficient depth (*e.g.* Jupiter’s moon Ganymede).

Furthermore, the manner in which we synthesize MS9 suggests another way in which this otherwise metastable hydrate could form and persist in nature. Salt hydrates on the surfaces of icy satellites may have been emplaced by cryovolcanic eruptions, perhaps involving production of droplets or spatter and perhaps with sufficient exsolution of volatiles to create a foam or fine spray. Under such circumstances it is easy to envisage the rapid chilling of fine droplets in the cold and very low-pressure environment (*ca* 100 K, few μPa) of an icy satellite’s surface leading to solidification of glassy beads, similar to the orange and green glassy volcanic spherules found in the lunar regolith (*e.g.* Heiken *et al.*, 1974 ▸). Annealing processes acting over gigayear timescales, or shorter periods in contact with ‘warm’ cryovolcanic deposits could conceivably lead to crystallization of MS9 and/or other as-yet unknown metastable cryohydrates from these glassy beads.

**Figure 12:**
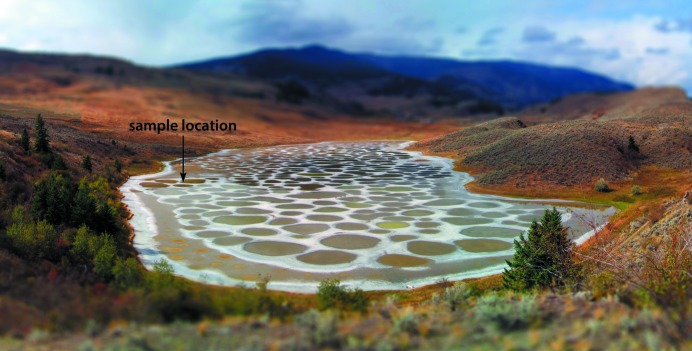
View of Spotted Lake looking north-west acquired by ADF in September 2013. The lake is ∼ 700 m long and 240 m across at its widest point, having an area of 13 hectares. The bulk of this endorheic basin is filled with coarse gypsum crystals and organic mud. Concentrated Mg^2+^–Na^+^–SO_4_^2−^ brine occupies some 670 discrete pools from < 1 m to ∼ 40 m across. The sample described in the text was obtained at 49° 4′ 36.98″ N, 119° 33′ 55.62″ W.

Lastly, it is possible that the presence of other solutes could ameliorate the growth and persistence of MS9, either by providing a nucleation template or by shifting the thermodynamics of the system in its favour. The possibility that this might occur was identified serendipitously: in broader work on MgSO_4_-rich hypersaline analogue environments, we had cause to collect brine samples on two occasions from Spotted Lake, near Osoyoos, British Columbia, Canada (Fig. 12 ▸).

The first sample was collected in late September (air temperature = 292 K), when the brine pools were precipitating large circumferential ‘reefs’ of water-clear mirabilite, thereby depleting the remaining liquid of sodium. The second sample was collected in late December of the same year (air temperature = 271 K), when the pools were roofed over with a layer of quite soft, wet ice ∼ 3 cm thick, beneath which was liquid. Brine samples were dried at 673 K for 24 h to determine the total dissolved solid content and the residue was then analysed for major elements using a Jeol JXA8100 microprobe. In both samples, the brine composition was dominated by Mg^2+^, Na^+^ and SO_4_^2−^ with very small quantities of K^+^ and Cl^−^ (∼ 0.5 and 0.2 mol L^−1^, respectively, in both samples). The autumn brine (concentration 26.6 wt %) had a Mg/Na ratio of 0.9, whereas in the winter brine (concentration 15.3 wt %) this had increased significantly to Mg/Na = 3.0, reflecting the precipitation of mirabilite throughout the summer and autumn. The remainder of the winter brine (∼ 30 g) was decanted into a plastic bottle and left in a chest freezer for several hours. At the end of this period the contents had solidified entirely. These were extracted, ground to a powder, and examined by X-ray powder diffraction on our Peltier cold stage.

**Figure 13:**
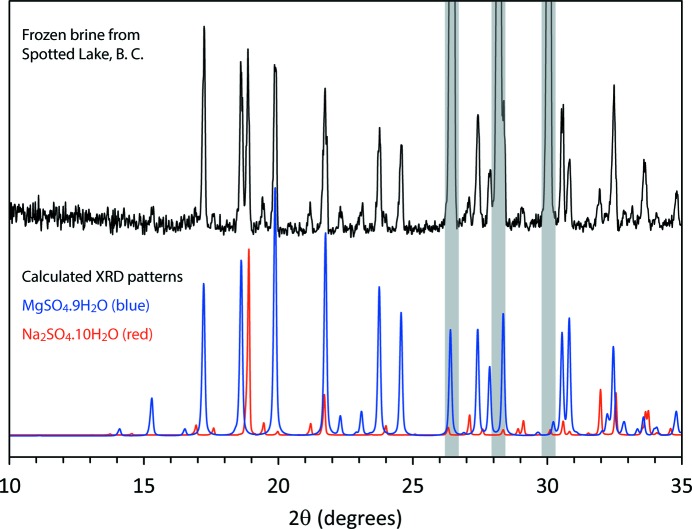
Top: X-ray powder diffraction data (λ = 1.788996 Å) from a slowly frozen specimen of winter sub-ice brine from Spotted Lake, British Columbia. Bottom: Calculated X-ray powder diffraction patterns of MS9 (blue) and mirabilite (red). Strong peaks from water ice are shaded grey. The phase fractions obtained by Rietveld refinement are 23.0 (5) wt % MgSO_4_·9H_2_O, 7.3 (5) wt % Na_2_SO_4_·10H_2_O, 69.7 (5) wt % ice.

Fig. 13 ▸ shows the diffraction pattern obtained from this specimen, which is clearly an excellent match for a mixture of MS9, mirabilite and water ice. Since our treatment of this brine is no different from what may occur during a cold snap at any of the MgSO_4_-rich lakes in British Columbia, we venture to suggest that MS9 forms in these environments, albeit on a transient basis, as a natural material. It also suggests that there may be a route to preparing single crystals by equilibrium growth from an appropriately doped aqueous solution. Testing the hypothesis that MS9 can occur naturally in cold hypersaline lakes requires collection, transport and analysis of frozen specimens ideally at liquid nitrogen temperatures or else *in situ* analysis with a portable X-ray diffractometer as long as the specimen is kept below 250 K during the measurement. *In situ* Raman spectroscopy would be the easiest method of identifying MS9 and work is in progress to characterize diagnostic differences between the vibrational spectra of MS7, MS9 and MS11. These efforts will pay dividends in future efforts to unambiguously identify cryohydrates by Raman spectroscopy done *in situ* on planetary surfaces and at very large stand-off distances, *i.e.* from orbit (Angel *et al.*, 2012 ▸).

It is worthwhile commenting on a recently published paper by Vu *et al.* (2016 ▸) in which various brines thought to be representative of the ocean composition of Jupiter’s icy satellite Europa were frozen and examined by Raman microscopic methods. These authors observed a prevalence of mirabilite over MgSO_4_ hydrates in all brine compositions, which ranged from Mg/Na ratios of 0.47 to 1.64. In the most Mg-rich frozen brines, Vu *et al.* report (their Fig. 5 ▸) a ν_1_ sulfate band at 990 cm^−1^, which they attribute solely to mirabilite. However, this band is quite asymmetric on the low-frequency side, as one would expect if there were a substantial unresolved shoulder near 980–985 cm^−1^ from a MgSO_4_ cryohydrate. Secondly, these authors could not be aware (reasonably) of either the existence or the Raman signature of a possible MgSO_4_·9H_2_O. Finally, their Mg/Na ratios are substantially smaller than the Spotted Lake sample from which we obtained MS9 + mirabilite, and are also smaller than the ratio predicted for a Europan ocean by the FREZCHEM Pitzer potential calculations of Marion *et al.* (2005 ▸). If nothing else, this work emphasizes (i) the value of establishing definitive vibrational signatures of these cryohydrates, since the internal sulfate modes do not differ substantially amongst the higher hydrates (Wang *et al.*, 2006 ▸) and so the less commonly measured low–frequency lattice modes or THz spectra are more likely to have interpretive value, and (ii) the importance of doing *simultaneous* diffraction and vibrational spectroscopy to provide certainty about the phases or phase mixtures being studied.

## 5. Concluding remarks   

We have determined the crystal structure of MgSO_4_·9H_2_O, including all hydrogen positions using a combination of X-ray and neutron powder diffraction. Additionally, we have determined the thermal expansion and incompressibility tensors of MgSO_4_·9H_2_O over the range 10–260 K at ambient pressure and 0–1 GPa at 240 K. Our observations have been instrumental in understanding the decomposition of meridianiite at high pressure and provide useful information on structural systematics and the transformation of metastable cryohydrates at low temperature. Further work is required to verify and further characterize an apparent elastic softening observed under compression at 240 K, but which is not reproduced in athermal *ab initio* calculations.

In future work we intend to characterize both the Zn^2+^ and Fe^2+^ analogues of MS9; since both of these have larger ionic radii than Mg^2+^ and different electronic structures, we expect to observe a difference in site substitution preferences and in the chemically induced strain of the unit cell. More importantly, we aim to provide the mineralogical and planetary science community with the spectroscopic data required to identify MS9 in the field – both on Earth and elsewhere.

## 6. Related literature   

References cited in the supporting information include: Alexandrov *et al.* (1963 ▸), Arbeck *et al.* (2010 ▸), Cook-Hallett *et al.* (2015 ▸), Fortes, Wood *et al.* (2003 ▸), Geruo *et al.* (2014 ▸), Gowen *et al.* (2011 ▸), Hill (1952 ▸), Nimmo (2004 ▸), Nimmo & Matsuyama (2007 ▸), Nimmo & Schenk (2006 ▸), Reuss (1929 ▸), Tsuji & Teanby (2016 ▸), Voigt (1910 ▸), Wahr *et al.* (2009 ▸).

## Supplementary Material

Crystal structure: contains datablock(s) 9_K_publ, 9_K_overall, 9_K_MS9, 9_K_ice, 9_K_p_01, 9_K_p_02, 9_K_p_04. DOI: 10.1107/S2052520616018266/fx5003sup1.cif

Structure factors: contains datablock(s) 9_K_p_01. DOI: 10.1107/S2052520616018266/fx50039_K_p_01sup2.hkl

Structure factors: contains datablock(s) 9_K_p_02. DOI: 10.1107/S2052520616018266/fx50039_K_p_02sup3.hkl

Structure factors: contains datablock(s) 9_K_p_04. DOI: 10.1107/S2052520616018266/fx50039_K_p_04sup4.hkl

DFT relaxed structure of MgSO4.9H2O using PBE GGA functional. DOI: 10.1107/S2052520616018266/fx5003sup5.txt

DFT relaxed structure of MgSO4.9H2O using Wu-Cohen GGA functional. DOI: 10.1107/S2052520616018266/fx5003sup6.txt

DFT relaxed structure of MgSO4.9H2O in the MgSeO4.9H2O structure (Wu-Cohen GGA). DOI: 10.1107/S2052520616018266/fx5003sup7.txt

Supplementary electronic PDF containing additional text, figures and tables. DOI: 10.1107/S2052520616018266/fx5003sup8.pdf

## Figures and Tables

**Table 1:** Unit-cell dimensions, bond lengths, angles and other geometric properties of the ionic polyhedra in MS9 compared with the zero-pressure DFT geometry optimizations The octahedral distortion index, quadratic elongation (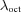) and bond-angle variance (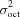) are defined by Robinson *et al.* (1971 ▸).

	MgSO_4_·9D_2_O (9 K exp)	MgSO_4_·9H_2_O (WC GGA)	MgSO_4_·9H_2_O (PBE GGA)
*a* (Å)	6.7273 (2)	6.63048	6.78924
*b* (Å)	11.9040 (2)	11.94593	12.19521
*c* (Å)	14.6405 (3)	14.63552	14.78586
β (°)	95.199 (1)	94.0222	94.3563
*V* (Å^3^)	1167.61 (2)	1156.3832	1220.6764
Δ*V* (%)	–	−0.96	+4.54
			
S—O1 (Å)	1.479 (4)	1.4911	1.4944
S—O2† (Å)	1.475 (4)	1.4719	1.4763
S—O3 (Å)	1.491 (4)	1.4948	1.4981
S—O4 (Å)	1.479 (4)	1.5063	1.5078
O1—S—O2 (°)	109.8 (3)	110.32	110.12
O1—S—O3 (°)	108.7 (3)	109.38	109.35
O1—S—O4 (°)	110.0 (3)	108.93	109.09
O2—S—O3 (°)	109.6 (3)	110.20	110.15
O2—S—O4 (°)	110.0 (3)	109.35	109.43
O3—S—O4 (°)	108.8 (3)	108.64	108.68
SO_4_ volume (Å^3^)	1.667	1.7009	1.7117
Δ*V* (%)	–	+2.0	+2.7
			
Mg1—O*w*1 (Å)	2.028 (5)	2.0546	2.0751
Mg1—O*w*2 (Å)	2.075 (5)	2.0946	2.1150
Mg1—O*w*3 (Å)	2.056 (5)	2.1320	2.1417
O*w*1—Mg1—O*w*2 (°)	90.3 (2)	90.24	90.26
O*w*1—Mg1—O*w*3 (°)	92.3 (2)	90.69	90.62
O*w*2—Mg1—O*w*3 (°)	91.4 (2)	93.80	93.35
Mg1 volume (Å^3^)	11.521	12.2054	12.5101
Δ*V* (%)	–	+5.9	+8.6
Dist. index	0.0080	0.0125	0.0112
λ_oct_	1.0009	1.0020	1.0015
σ^2^_oct_ (°^2^)	2.6586	5.4403	4.2356
			
Mg2—O*w*4 (Å)	2.034 (4)	2.0696	2.0877
Mg2—O*w*5‡ (Å)	2.108 (5)	2.1307	2.1448
Mg2—O*w*6 (Å)	2.051 (4)	2.0858	2.1019
O*w*4—Mg2—O*w*5 (°)	90.1 (2)	90.02	90.60
O*w*4—Mg2—O*w*6 (°)	90.2 (2)	91.28	90.94
O*w*5—Mg2—O*w*6 (°)	90.6 (2)	90.89	91.08
Mg2 volume (Å^3^)	11.725	12.2585	12.5439
Δ*V* (%)	–	+4.0	+7.0
Dist. index	0.0142	0.0113	0.0105
λ_oct_	1.0005	1.0005	1.0005
σ^2^_oct_ (°^2^)	0.1396	0.8798	0.8770

† Accepts two hydrogen bonds (all others accept three).

‡ Hydrogen-bond acceptor in tetrahedral coordination.

**Table 2:** Hydrogen-bond lengths and angles in MS9 at 9 K

	D—O—D (°)	O—D (Å)	D⋯O (Å)	O⋯O (Å)	O—D⋯O (°)
O*w*1—D1*a*⋯O2	106.5 (6)	0.968 (4)	1.761 (6)	2.699 (6)	162.3 (5)
O*w*1—D1*b*⋯O*w*9		0.970 (4)	1.811 (6)	2.775 (6)	171.9 (5)
O*w*2—D2*a*⋯O*w*5	103.8 (6)	0.971 (4)	2.051 (7)	3.014 (7)	171.0 (5)
O*w*2—D2*b*⋯O*w*9		0.974 (4)	1.834 (7)	2.786 (7)	164.9 (5)
O*w*3—D3*a*⋯O*w*8	102.2 (6)	0.978 (4)	1.880 (7)	2.855 (6)	175.8 (5)
O*w*3—D3*b*⋯O1		0.964 (4)	1.933 (6)	2.894 (6)	174.4 (5)
O*w*4—D4*a*⋯O*w*7	106.5 (6)	0.969 (4)	1.784 (6)	2.734 (6)	165.6 (5)
O*w*4—D4*b*⋯O1		0.976 (4)	1.822 (6)	2.797 (6)	176.7 (5)
O*w*5—D5*a*⋯O*w*7	107.1 (6)	0.980 (4)	1.695 (7)	2.671 (7)	173.6 (6)
O*w*5—D5*b*⋯O4		0.971 (4)	1.753 (6)	2.723 (6)	176.4 (5)
O*w*6—D6*a*⋯O*w*8	106.1 (6)	0.982 (4)	1.771 (7)	2.715 (6)	160.3 (5)
O*w*6—D6*b*⋯O3		0.957 (4)	1.774 (6)	2.722 (6)	170.5 (5)
O*w*7—D7*a*⋯O3	103.8 (6)	0.989 (4)	1.793 (6)	2.859 (6)	164.6 (5)
O*w*7—D7*b*⋯O2		0.988 (4)	1.750 (6)	2.730 (6)	171.7 (5)
O*w*8—D8*a*⋯O4	103.7 (6)	0.992 (4)	1.882 (6)	2.847 (6)	163.5 (5)
O*w*8—D8*b*⋯O1		0.985 (4)	1.942 (6)	2.925 (6)	175.1 (5)
O*w*9—D9*a*⋯O3	102.1 (6)	0.972 (4)	1.859 (6)	2.813 (6)	166.4 (5)
O*w*9—D9*b*⋯O4		0.979 (4)	1.792 (6)	2.766 (6)	172.6 (5)

**Table 3:** Einstein fit parameters [equations (4) and (6) for the volume, (5) and (6) for cell parameters]

	*a*-axis	*b*-axis	*c*-axis	β	*V*
*X*_0_ (Å, Å^3^)	6.7274 (1)	11.9039 (1)	14.6404 (1)	95.199 (1)	1167.63 (1)
θ (K)	189 ± 15	153 ± 7	439 ± 14	59 ± 15	209 ± 5
*E*_0_	1.8 (5) × 10^−2^	2.9 (3) × 10^−2^	2.5 (3) × 10^−1^	−1.4 (4) × 10^−2^	12.5 (8)
*E*_1_	1.10 (4) × 10^−4^	1.25 (2) × 10^−4^	−2.7 (7) × 10^−4^	–	4.21 (7) × 10^−2^

**Table 4:** Murnaghan integrated linear equation of state [equation (6)] fit parameters

	*a*-axis	*b*-axis	*c*-axis	β	*V*
MILEOS parameters including PEARL/HiPr datum (240 K)					
*X*_0_	6.7701 (7)	11.9705 (9)	14.6799 (4)	95.106 (6)	1184.8 (2)
*K*_0_	51 (2)	39.9 (6)	139 (2)	−132 (8)	19.5 (3)
*K*′	40 (3)	10 (1)	−60 (3)	−292 (15)	3.8 (4)
					
MILEOS parameters excluding PEARL/HiPr datum (240 K)					
*X*_0_	6.7694 (6)	11.9708 (12)	14.6801 (5)	95.112 (3)	1184.9 (2)
*K*_0_	55 (3)	39 (1)	135 (5)	−154 (7)	19.3 (6)
*K*′	24 (10)	12 (5)	−44 (18)	−195 (27)	4 (2)
					
MILEOS parameters (athermal WC GGA calculations)					
*X*_0_	6.625 (1)	11.963 (4)	14.633 (2)	94.12 (2)	1156.9 (4)
*K*_0_	61 (1)	58 (1)	140 (2)	−287 (17)	24.2 (2)
*K*′	17 (1)	7 (1)	15 (2)	−49 (16)	4.1 (2)

**Table 5:** Derived linear and volume compressibilities (*B*) and incompressibilities (*K*) found by fitting linear expressions to the pressure dependences of the elastic strain tensor’s eigenvalues (HRPD + PEARL/HiPr) Values in italics are those found from DFT calculations (athermal, WC GGA).

	Compressibility		Incompressibility	
	*B*_0_ (GPa^−1^)	*B*′	*K*_0_ (GPa)	*K*′
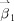	2.24 × 10^−2^	−1.92 × 10^−2^	44.6	40.9
	*1.75 × 10^−2^*	*−4.6 × 10^−3^*	*57.3*	*14.9*
				
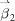	2.50 × 10^−2^	−6.0 × 10^−3^	40.0	9.8
	*1.72 × 10^−2^*	*−1.9 × 10^−3^*	*58.2*	*6.5*
				
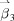	5.2 × 10^−3^	7.1 × 10^−3^	190.8	−234.0
	*6.5 × 10^−3^*	*−8.0 × 10^−4^*	*153.4*	*18.8*
				
β_V_	5.27 × 10^−2^	−1.81 × 10^−2^	19.0	6.7
	*4.12 × 10^−2^*	*−7.3 × 10^−3^*	*24.3*	*4.3*

## References

[bb1] Alexandrov, K. S., Rhyzhova, T. V. & Rostuntseva, A. I. (1963). *Soviet Phys. Cryst.* **7**, 753–755.

[bb2] Angel, S. M., Gomer, N. R., Sharma, S. K. & McKay, C. (2012). *Appl. Spectrosc.* **66**, 137–150. 10.1366/11-06535 22449277

[bb3] Arbeck, D., Haussühl, E., Bayarjagal, L., Winkler, B., Paulsen, N., Haussühl, S. & Milman, V. (2010). *Eur. Phys. J. B*, **73**, 167–175.

[bb5] Avrami, M. (1939). *J. Chem. Phys.* **7**, 1103–1112.

[bb6] Avrami, M. (1940). *J. Chem. Phys.* **8**, 212–224.

[bb8] Boisen, M. B. Jr & Gibbs, G. V. (1990). *Rev. Mineral.* **15**, 72–75.

[bb9] Brand, H. E. A., Fortes, A. D., Wood, I. G., Knight, K. S. & Vočadlo, L. (2009). *Phys. Chem. Miner.* **36**, 29–46.

[bb10] Brand, H. E. A., Fortes, A. D., Wood, I. G. & Vočadlo, L. (2010). *Phys. Chem. Miner.* **37**, 265–282.

[bb11] Brown, M. E. & Hand, K. P. (2013). *Astron. J.* **145**, 110.

[bb12] Cannon, K. M. (2012). PhD Thesis. Queen’s University, Ontario.

[bb13] Chipera, S. J. & Vaniman, D. T. (2007). *Geochim. Cosmochim. Acta*, **71**, 241–250.

[bb14] Chou, I. & Seal, R. R. (2003). *Astrobiology*, **3**, 619–630. 10.1089/153110703322610708 14678670

[bb15] Clark, B. C., Baird, A. K., Rose, H. J., Toulmin, P., Keil, K., Castro, A. J., Kelliher, W. C., Rowe, C. D. & Evans, P. H. (1976). *Science*, **194**, 1283–1288. 10.1126/science.194.4271.1283 17797084

[bb16] Clark, S. J., Segall, M. D., Pickard, C. J., Hasnip, P. J., Probert, M. I. J., Refson, K. & Payne, M. C. (2005). *Z. Kristallogr.* **220**, 567–570.

[bb17] Cook-Hallett, C., Barnes, J. W., Kattenhorn, S. A., Hurford, T., Radebaugh, J., Stiles, B. & Beuthe, M. (2015). *J. Geophys. Res. Planets*, **120**, 1220–1236.

[bb18] Dalton, J. B. (2007). *Geophys. Res. Lett.* **34**, L21205.

[bb19] Dalton, J. B., Prieto-Ballesteros, O., Kargel, J. S., Jamieson, C. S., Jolivet, J. & Quinn, R. (2005). *Icarus*, **177**, 472–490.

[bb20] D’Ans, J. (1933). *Die lösungsgleichgewichte der system der salz ozeanischer salzablagerungen*, pp. 118–123. Berlin: Verlagsgesellschaft für Ackerban.

[bb21] Favre-Nicolin, V. & Černý, R. (2002). *J. Appl. Cryst.* **35**, 734–743.

[bb22] Favre-Nicolin, V. & Černý, R. (2004). *Z. Kristallogr.* **219**, 847–856.

[bb23] Feldman, W. C., Mellon, M. T., Maurice, S., Prettyman, T. H., Carey, J. W., Vaniman, D. T., Bish, D. L., Fialips, C. I., Chipera, S. J., Kargel, J. S., Elphic, R. C., Funsten, H. O., Lawrence, D. J. & Tokar, R. L. (2004). *Geophys. Res. Lett.* **31**, L16702.

[bb24] Feldman, W. C., Prettyman, T. H., Maurice, S., Plaut, J. J., Bish, D. L., Vaniman, D. T., Mellon, M. T., Metzger, A. E., Squyres, S. W., Karunatillake, S., Boynton, W. V., Elphic, R. C., Funsten, H. O., Lawrence, D. J. & Tokar, R. L. (2004). *J. Geophys. Res. Planets.* **109**, E09006.

[bb25] Foley, C. N., Economou, T. & Clayton, R. N. (2003). *J. Geophys. Res.* **108**, 8096.

[bb26] Fortes, A. D. (2015). *Powder Diffr.* **30**, 149–157.

[bb27] Fortes, A. D. (2016). ISIS Experimental Report RB 1520149. Rutherford Appleton Laboratory, Chilton, UK.

[bb28] Fortes, A. D., Alfè, D., Hernández, E. R. & Gutmann, M. J. (2015). *Acta Cryst.* B**71**, 313–327. 10.1107/S2052520615006824 PMC4450603 26027007

[bb29] Fortes, A. D., Browning, F. & Wood, I. G. (2012*a*). *Phys. Chem. Miner.* **39**, 419–441.

[bb30] Fortes, A. D., Browning, F. & Wood, I. G. (2012*b*). *Phys. Chem. Miner.* **39**, 443–454.

[bb31] Fortes, A. D., Fernandez-Alonso, F., Tucker, M. G. & Wood, I. G. (2017). *Acta Cryst.* B**73**, 33–46.

[bb32] Fortes, A. D. & Wood, I. G. (2012). *Powder Diffr.* **27**, 8–11.

[bb35] Fortes, A. D., Wood, I. G., Alfé, D., Hernàndez, E. R., Gutmann, M. J. & Sparkes, H. A. (2014). *Acta Cryst.* B**70**, 948–962. 10.1107/S205252061402126X PMC4468514 25449618

[bb33] Fortes, A. D., Wood, I. G., Alfredsson, M., Vočadlo, L. & Knight, K. S. (2006). *Eur. J. Mineral.* **18**, 449–462.

[bb34] Fortes, A. D., Wood, I. G., Brodholt, J. P., Alfredsson, M., Vocadlo, L., McGrady, G. S. & Knight, K. S. (2003). *J. Chem. Phys.* **119**, 10806–10813.

[bb36] Fortes, A. D., Wood, I. G. & Knight, K. S. (2008). *Phys. Chem. Miner.* **35**, 207–221.

[bb37] Fortes, A. D., Wood, I. G., Tucker, M. G. & Marshall, W. G. (2012). *J. Appl. Cryst.* **45**, 523–534.

[bb38] Genceli, F. E., Horikawa, S., Iizuka, Y., Sakurai, T., Hondoh, T., Kawamura, T. & Witkamp, G. (2009). *J. Glaciology*, **55**, 117–122.

[bb39] Geruo, A., Wahr, J. & Zhong, S. (2014). *J. Geophys. Res. Planets*, **119**, 659–678.

[bb40] Gowen, R. A. *et al.* (2011). *Adv. Space Res.* **48**, 725–742.

[bb41] Gromnitskaya, E. L., Yagafarov, O. F., Lyapin, A. G., Brazhkin, V. V., Wood, I. G., Tucker, M. G. & Fortes, A. D. (2013). *Phys. Chem. Miner.* **40**, 271–285.

[bb42] Haas, P., Tran, F. & Blaha, P. (2009). *Phys. Rev. B*, **79**, 085104.

[bb43] Haas, P., Tran, F., Blaha, P. & Schwarz, K. (2011). *Phys. Rev. B*, **83**, 205117.

[bb44] Hashash, Y. M. A., Yao, J. I. & Wotring, D. C. (2003). *Int. J. Numer. Anal. Methods Geomech.* **27**, 603–626.

[bb45] Hazen, R. M. & Finger, L. W. (1982). *Comparative Crystal Chemistry.* New York: John Wiley and Sons.

[bb46] Heiken, G. H., McKay, D. S. & Brown, R. W. (1974). *Geochim. Cosmochim. Acta*, **38**, 1703–1718.

[bb47] Hennings, E., Schmidt, H. & Voigt, W. (2013). *Acta Cryst.* C**69**, 1292–1300. 10.1107/S0108270113028138 24192174

[bb48] Hennings, E., Schmidt, H. & Voigt, W. (2014*a*). *Acta Cryst.* E**70**, 477–479. 10.1107/S1600536814024295 PMC4257414 25552970

[bb50] Hennings, E., Schmidt, H. & Voigt, W. (2014*b*). *Acta Cryst.* C**70**, 876–881. 10.1107/S2053229614018002 25186361

[bb51] Hill, R. (1952). *Proc. Phys. Soc. London*, **65**, 349–354.

[bb52] Hodenberg, R. & Kühn, R. (1967). *Kali. Steinsalz* **4**, 326–340.

[bb53] Hohenberg, P. & Kohn, W. (1964). *Phys. Rev. B*, **136**, 864–871.

[bb54] Ibberson, R. M. (2009). *Nucl. Instrum. Methods Phys. Res. A*, **600**, 47–49.

[bb55] Ibberson, R. M., David, W. I. F. & Knight, K. S. (1992). *The High Resolution Neutron Powder Diffractometer (HRPD) at ISIS – a User Guide.* RAL-92–031. Rutherford Appleton Laboratory, Chilton, UK, http://www.isis.stfc.ac.uk/instruments/hrpd/documents/hrpd-manual6735.pdf.

[bb56] International Association for the Properties of Water & Steam, IAPWS (2011). Revised release on the pressure along the melting and sublimation curves of ordinary water substance, http://www.iapws.org.

[bb58] Johnson, W. A. & Mehl, R. F. (1939). *Trans. AIME* **135**, 416–442 (Tech. Pub. No. 1089).

[bb59] Kaminsky, W. (2004). *WinTensor*1.1. University of Washington, USA, http://cad4.cpac.washington.edu/WinTensorhome/WinTensor.htm.

[bb60] Kaminsky, W. (2007). *J. Appl. Cryst.* **40**, 382–385.

[bb61] Kargel, J. S. (1991). *Icarus*, **94**, 368–390.

[bb62] Khurana, K. K., Kivelson, M. G., Stevenson, D. J., Schubert, G., Russell, C. T., Walker, R. J. & Polanskey, C. (1998). *Nature*, **395**, 777–780. 10.1038/27394 9796812

[bb63] King, P. L., Lescinsky, D. T. & Nesbitt, H. W. (2004). *Geochim. Cosmochim. Acta*, **68**, 4993–5008.

[bb64] Kivelson, M. G., Khurana, K. K. & Volwerk, M. (2002). *Icarus*, **157**, 507–522.

[bb65] Kohn, W. & Sham, L. J. (1965). *Phys. Rev.* **140**, A1133–A1138.

[bb66] Kolmogorov, A. N. (1937). *Izv. Akad. Nauk. SSSR Ser. Mater.* **3**, 355–359.

[bb68] Larsen, A. C. & Von Dreele, R. B. (2000). *GSAS.* Report LAUR 86–748. Los Alamos National Laboratory, New Mexico, USA, https://www.ncnr.nist.gov/Xtal/software/gsas.html.

[bb69] Marion, G. M., Kargel, J. S., Catling, D. C. & Jakubowski, S. D. (2005). *Geochim. Cosmochim. Acta*, **69**, 259–274.

[bb70] Matsuo, S., Kuniyoshi, H. & Miyake, Y. (1964). *Science*, **145**, 1454–1455. 10.1126/science.145.3639.1454 17838707

[bb71] McSween, H. Y. (2004). *Science*, **305**, 842–845.

[bb74] Murnaghan, F. D. (1944). *Proc. Natl Acad. Sci. USA*, **30**, 244–247. 10.1073/pnas.30.9.244 PMC1078704 16588651

[bb75] Nimmo, F. (2004). *J. Geophys. Res.* **109**, E12001, http://dx.doi.org/10.1029/2004JE002347.

[bb76] Nimmo, F. & Matsuyama, I. (2007). *Geophys. Res. Lett.* **34**, L19203, http://dx.doi.org/10.1029/2007GL030798.

[bb77] Nimmo, F. & Schenk, P. (2006). *J. Struct. Geol.* **28**, 2194–2203.

[bb78] Orlando, T. M., McCord, T. B. & Grieves, G. A. (2005). *Icarus*, **177**, 528–533.

[bb80] Payne, M. C., Teter, M. P., Allan, D. C., Arias, T. A. & Joannopoulos, J. D. (1992). *Rev. Mod. Phys.* **64**, 1045–1097.

[bb81] Perdew, J. P., Burke, K. & Ernzerhof, M. (1996). *Phys. Rev. Lett.* **77**, 3865–3868. 10.1103/PhysRevLett.77.3865 10062328

[bb82] Perdew, J. P., Burke, K. & Ernzerhof, M. (1997). *Phys. Rev. Lett.* **78**, 1396. 10.1103/PhysRevLett.77.3865 10062328

[bb83] Peterson, R. C., Nelson, W., Madu, B. & Shurvell, H. F. (2007). *Am. Mineral.* **92**, 1756–1759.

[bb84] Peterson, R. C. & Wang, R. (2006). *Geology*, **34**, 957–960.

[bb85] Pfrommer, B. G., Côté, M., Louie, S. G. & Cohen, M. L. (1997). *J. Comput. Phys.* **131**, 233–240.

[bb86] Pistorius, C. W. F. T. (1968). *J. Chem. Phys.* **48**, 5509–5514.

[bb87] POV (2004). *Persistence of Vision Raytracer*, Version 3.6. Persistence of Vision Pty Ltd, http://www.povray.org/.

[bb88] Putz, H. & Brandenburg, K. (2006). *Diamond.* Crystal Impact GbR, Bonn, Germany, http://www.crystalimpact.com/diamond.

[bb89] Robinson, K., Gibbs, G. V. & Ribbe, P. H. (1971). *Science*, **172**, 567–570. 10.1126/science.172.3983.567 17802221

[bb90] Reuss, A. (1929). *Z. Angew. Math. Mech.* **9**, 49–58.

[bb91] Sakurai, T., Iizuka, Y., Horikawa, S., Johnsen, S., Dahl-Jensen, D., Steffensen, J. P. & Hondoh, T. (2009). *J. Glaciology*, **55**, 777–783.

[bb92] Schlenker, J. L., Gibbs, G. V. & Boisen, M. B. (1978). *Acta Cryst.* A**34**, 52–54.

[bb93] Schmidt, H., Hennings, E. & Voigt, W. (2014). *Acta Cryst.* C**70**, 882–888. 10.1107/S2053229614014302 25186362

[bb94] Segall, M. D., Lindan, P. J. D., Probert, M. J., Pickard, C. J., Hasnip, P. J., Clark, S. J. & Payne, M. C. (2002). *J. Phys. Condens. Matter*, **14**, 2717–2744.

[bb95] Shirley, J. H., Dalton, J. B., Prockter, L. M. & Kamp, L. W. (2010). *Icarus*, **210**, 358–384.

[bb96] Toby, B. H. (2001). *J. Appl. Cryst.* **34**, 210–213.

[bb97] Toulmin, P., Baird, A. K., Clark, B. C., Keil, K., Rose, H. J., Christian, R. P., Evans, P. H. & Kelliher, W. C. (1977). *J. Geophys. Res.* **82**, 4625–4634.

[bb98] Tran, F., Laskowski, R., Blaha, P. & Schwarz, K. (2007). *Phys. Rev. B*, **75**, 115131.

[bb99] Tsuji, D. & Teanby, N. A. (2016). *Icarus*, **277**, 39–55.

[bb100] Vance, S., Bouffard, M., Choukroun, M. & Sotin, C. (2014). *Planet. Space Sci.* **96**, 62–70.

[bb101] Vance, S. & Brown, J. M. (2013). *Geochim. Cosmochim. Acta*, **110**, 176–189.

[bb102] Voigt, W. (1910). *Lehrbuch der Kristallphysik.* Leipzig: Taubner.

[bb103] Vu, T. H., Hodyss, R., Choukroun, M. & Johnson, P. V. (2016). *ApJ*, **816**, L26.

[bb104] Wagner, W., Riethmann, T., Feistel, R. & Harvey, A. H. (2011). *J. Phys. Chem. Ref. Data*, **40**, 043103.

[bb105] Wahr, J., Selvans, Z. A., Mullen, M. E., Barr, A. C., Collins, G. C., Selvans, M. M. & Pappalardo, R. T. (2009). *Icarus*, **200**, 188–206.

[bb106] Wang, A., Freeman, J. J., Chou, I.-M. & Jolliff, B. L. (2011). *J. Geophys. Res.* **116**, E12006.

[bb107] Wang, A., Freeman, J. J. & Jolliff, B. L. (2009). *J. Geophys. Res.* **114**, E04010.

[bb108] Wang, A., Freeman, J. J., Jolliff, B. L. & Chou, I.-M. (2006). *Geochim. Cosmochim. Acta*, **70**, 6118–6135.

[bb109] Wänke, H., Brückner, J., Dreibus, G., Rieder, R. & Ryabchikov, I. (2001). *Space Sci. Rev.* **96**, 317–330.

[bb110] Wood, I. G., Hughes, N. J., Browning, F. & Fortes, A. D. (2012). *J. Appl. Cryst.* **45**, 608–610.

[bb111] Wu, Z. & Cohen, R. E. (2006). *Phys. Rev. B*, **73**, 235116.

