# Supplementary material for: Structure, thermal expansion and incompressibility of MgSO4·9H2O, its relationship to meridianiite (MgSO4·11H2O) and possible natural occurrences
Source: Acta Crystallogr B Struct Sci Cryst Eng Mater. 2017 Jan 31;73(Pt 1):47–64. doi: 10.1107/S2052520616018266 (PMC5289306; doi:10.1107/S2052520616018266)
Supplement: Supplementary file 8 [file b-73-00047-sup8.pdf]

S U P P L E M E N T A R Y   M A T E R I A L

Structure, thermal expansion and incompressibility of $MgSO_4 \cdot 9H_2O$, its relationship to meridianiite ($MgSO_4 \cdot 11H_2O$) and possible natural occurrences.

A. D. Fortes[1,2*], K. S. Knight[1,2,3] and I. G. Wood[2]

[1]*ISIS Facility, STFC Rutherford Appleton Laboratory, Harwell Science and Innovation Campus, Chilton, Didcot, Oxfordshire OX11 0QX, UK.*

[2]*Department of Earth Sciences, University College London, Gower Street, London WC1E 6BT, UK.*

[3]*The Natural History Museum, Cromwell Road, London SW7 5BD, UK.*

*corresponding author (dominic.fortes@stfc.ac.uk)

CONTENTS

Supplementary text

1. Extended Debye-model analysis of the thermal expansion of $MgSO_4 \cdot 9H_2O$.

2. Calculation of the elastic constants of $MgSO_4 \cdot 9H_2O$ and other derived quantities.

Supplementary Figures

Figure S1: Neutron powder diffraction pattern of $MgSO_4 \cdot 11D_2O$, revealing accessory $MgSO_4 \cdot 9H_2O$ for the first time.

Figure S2 X-ray powder diffraction patterns from cation-pure MS9 (protonated and deuterated) compared with mixed $(Mg,Ni)SO_4 \cdot 9H_2O$.

Figure S3 Trial structure of MS9 with difference Fourier revealing three interstitial water molecules.

Figure S4 Illustration of the hexamer and trimer chains on H-bonded water in the structure of $MgSO_4 \cdot 9D_2O$.

Figure S5 Time-dependent X-ray powder data illustrating the reaction, MS9 + ice → MS11.

Figure S6 Time-dependent neutron powder data illustrating the reaction, MS9 + ice → MS11.

Figure S7 X-ray powder diffraction data showing the partial dehydration of MS9 over 12 hr.

Supplementary Tables

Table S1: Cell parameters of MS9 as a function of temperature

Table S2: Cell parameters of MS9 as a function of pressure

Table S3: Fit parameters from extended Debye model fitting to the thermal expansion of MS9.

Table S4: Calculated athermal elastic constants of MS9.

Table S5: Properties of MS9 derived from the calculated elastic constants.

References

Citations relating only to text and captions in this supplement.

Supplementary text

1. Extended Debye-model analysis of the thermal expansion

The Einstein model formalism described in the main text is not as accurate as the more rigorous Debye model, which would be the preferred choice when heat capacity data are available to aid in constraining the Debye temperatures. This is a particular problem for these types of molecular materials where models invoking two (or more) characteristic Debye temperatures are necessary and one is also obliged to determine the relative contributions of these terms to the internal energy. A first-order double-Debye model uses six variable parameters (versus four in the Einstein model); in practice it is impossible to determine these uniquely from thermal expansion data alone. It is nevertheless interesting to attempt a restrained fit of such models to V(T) in order to see what information might be obtained.

For a single-Debye model the temperature dependence of the volume is described as:

$$V(T) = V_0\left(1 + \frac{U(T)}{Q}\right) \qquad \text{(Eq. S1)}$$

where $Q = (V_0 K_T / \gamma)$ and V_0, K_T and γ have the same meaning as previously given. The internal energy of the crystal, U(T), is calculated via the Debye approximation for the heat capacity,

$$\mathrm{U}(\mathrm{T}) = 9\mathrm{Nk_B T}\left(\frac{\mathrm{T}}{\theta_D}\right)^3 \int_0^{\theta_D/\mathrm{T}} \frac{x^3}{e^x - 1}dx \qquad \text{(Eq. S2)}$$

where θ_D is the characteristic Debye temperature, N is the number of atoms per formula unit, k_B is the Boltzmann constant, and $x = \hbar\omega/k_BT$. Note that the vibrational zero-point energy of $9Nk_B\theta_D/8$ is included via the term V_0. This model, with three adjustable parameters, V_0, Q and θ_D, is fitted to the observed V(T) to give the values listed in Supplementary Table S3.

However, it is clear (as expected) that there is a significant temperature-dependent residual when using a single Debye temperature. Consequently, we adopt a model in which there are two adjustable Debye temperatures:

$$\mathrm{V(T)} = \mathrm{V_0}\left(1 + \frac{x\mathrm{U(T)}_1 + y\mathrm{U(T)}_2}{\mathrm{Q}}\right) \qquad \text{(Eq. S3)}$$

where $U(T)_1$ and $U(T)_2$ represent two distinct vibrational contributions to the internal energy of the crystal with characteristic high-frequency cut-offs defined by two Debye temperatures, θ_D^1 and θ_D^2: the magnitude of their relative contributions is given by the mixing parameters *x* and *y*. Ordinarily, one would fit the molar heat capacity of the material in order to obtain accurate values of the Debye temperatures, and particularly of the two mixing parameters, since these are quite poorly determined from thermal expansion data. In this instance, we found that it was necessary to fix V_0 at the observed 9 K value, 175.7872 cm^3 mol^{-1}, and also to fix K_T (and thus Q, assuming $\gamma = 1$) at the value found in the high-pressure part of this work – see Section 3.4), 19.50 GPa. This allows us to obtain tolerably well-determined values of the two Debye temperatures and the mixing parameters. It is worth observing that the sum $x + y = 0.64$, which implies that the heat capacity of MS9 does not approach the classical high temperature limit of Dulong and Petit ($3Nk_B$), but instead tends to $2Nk_B$. This is remarkably similar to the result we obtained from fitting the thermal expansion of ammonia dihydrate (Fortes *et al.,* 2003) and, in both cases, probably reflects the fact that neither substance can reach its high-temperature limit by virtue either of melting (at 175 K for ammonia dihydrate) or transforming to another phase (at 260 K for MS9).

Figure 6d compares the volume thermal expansion coefficient obtained from the modified Einstein and the double-Debye models: these clearly diverge above 220 K so we have fitted the Einstein-derived V(T) with the double-Debye model to evaluate the source of the difference. As

shown in Supplementary Table S3, the main difference is in a larger contribution from a much higher θ_D^2 and $x + y = 0.95$, which *is* now consistent with the high-temperature Dulong-Petit limit ($x + y = 1$). Indeed these values are quite similar in many respects to those used in analyzing the thermal expansion of MS7 (Fortes *et al.*, 2006), which had been derived from fitting the heat capacity of $ZnSO_4 \cdot 7H_2O$, where $\theta_D^1 = 238 \pm 5$ K, $\theta_D^2 = 1166 \pm 6$ K, $x = 0.26(1)$ and $y = 0.60(1)$.

The values of θ_D^1 and θ_D^2 given in Supplementary Table S3 are equivalent to vibrational frequencies of 244 and 1034 cm^{-1}, which almost certainly correspond to the high-frequency cut-offs of the translational and rotational librations of water, respectively, so it is not surprising that these should be similar in a range of hydrates with interstitial water molecules.

It is reassuring that a model with a bulk modulus matching the experimentally-determined value fits the zero-pressure thermal expansion so well.

---

2. Calculated elastic constants and other derived properties of MS9

The hydrostatic compression calculations described in the main text effectively provide (in the form of the elastic strain tensor) various linear sums of elastic compliances, s_{ij}; $e_1 = (s_{11} + s_{12} + s_{13})$, $e_2 = (s_{12} + s_{22} + s_{23})$, $e_3 = (s_{13} + s_{23} + s_{33})$ and $e_5 = (s_{15} + s_{25} + s_{35})$. Hence only nine of the thirteen independent 2nd-order elastic moduli are 'sampled', those excluded being s_{44}, s_{46}, s_{55} and s_{66}. In the absence of single crystals and given the metastability of this phase and the requirement to handle it at very low temperatures, the prospect of obtaining experimental data on the individual elastic constants seems remote. We have therefore used CASTEP to compute the complete set of thirteen elastic stiffness moduli from first principles.

This was achieved by applying a series of strains of varying amplitudes, the general strain matrix taking the form

$$\vec{\varepsilon} = \begin{pmatrix} \varepsilon_1 & \varepsilon_6/2 & \varepsilon_5/2 \\ \varepsilon_6/2 & \varepsilon_2 & \varepsilon_4/2 \\ \varepsilon_5/2 & \varepsilon_4/2 & \varepsilon_3 \end{pmatrix} \qquad \text{(Eq. S4)}$$

with the four independent sets of applied strains being (δ, 0, 0, δ, 0, 0); (0, 0, δ, 0, 0, δ); (0, δ, 0, 0, 0, 0); and (0, 0, 0, 0, δ, 0). The value of δ was varied between ± 0.03 in eight increments; at each strain amplitude, the ions were relaxed and a stress tensor computed, resulting in a total of thirty-

three calculated 3x3 stress tensors. The elastic constants are then simply partial derivatives of the stress with respect to the applied strain evaluated at zero strain:

$$c_{ij} = \left(\frac{\partial \sigma_i}{\partial e_j}\right)_0 \qquad \text{(Eq. S5)}$$

The results of the calculations were fitted with second-order polynomials to obtain c_{ij} and their uncertainties; the elastic compliances and their propagated uncertainties were then found by matrix inversion, $s_{ij} = c_{ij}^{-1}$. The same method was employed by Arbeck *et al.* (2010) to obtain accurate (if not so precise) elastic constants for the tetragonal polymorph of $NiSO_4 \cdot 6H_2O$ (retgersite). Supplementary Table S4 lists the calculated c_{ij} and s_{ij} of MS9 along with elastic constants (c_{ij}) measured by Alexandrov *et al.* (1963) for a number of orthorhombic and monoclinic heptahydrates of divalent metal sulfates. After the bulk and directional elastic properties for MS9 reported in the accompanying paper, these offer the best available experimental data against which to check that the DFT elastic constants are accurate. Indeed, with the exception of the larger c_{33} in MS9, which is supported observationally by the largest eigenvalue of the compressibility tensor and the smallest eigenvalue of the thermal expansion tensor being closely aligned to the *c*-axis, there is a high degree of similarity between the elastic constants of MS9 and the tabulated heptahydrates. The polyhedral units in these materials are substantially stiffer than the bulk crystal: in MS9 the calculated polyhedral bulk moduli are (*i*) 236 ± 5 GPa for the SO_4 tetrahedra; (*ii*) 91 ± 4 GPa for the M1 octahedral site; and (*iii*) 60 ± 1 GPa for the M2 octahedral site. This means that the overall elastic properties of the material are dominated by the intermolecular hydrogen bonds and the similarities in the elastic constants between the 9- and 7-hydrates are thus not surprising. Since both $MgSO_4 \cdot 11H_2O$ and $MgSeO_4 \cdot 9H_2O$ may be grown very easily as large single crystals, it would be instructive to measure their elastic constants.

From the calculated elastic moduli we obtain a number of other useful quantities. The bulk elastic properties of a randomly oriented aggregate of single crystals may be calculated for two cases, one in which all components of the aggregrate are assumed to be experiencing the same strain (the Voigt upper bound: Voigt, 1910) and one in which all components are assumed to be subject to the same stress (the Reuss lower bound: Reuss, 1929). The bulk modulus (K) and shear modulus (G) determined from the Voigt limits (Eq. S6 and S7) and from the Reuss limits (Eq. S8 and S9) are given in Supplementary Table S5. As determined by Hill (1952) an accurate estimate of an *anisotropic* crystalline aggregrate's bulk elastic properties is given by the arithmetical mean of the Voigt and Reuss bounds, which is the so-called Voigt-Reuss-Hill average (Eq. S10 and S11).

Other polycrystalline averages, such as Young's modulus, E (Eq. S12), Poisson's ratio, η (Eq. S13) and the longitudinal and transverse wave speeds, V_P and V_S (Eqs. S14 and S15). These last two are of particular relevance since they represent quantities that can (and should) be measured ultrasonically both under ambient conditions and as a function of pressure and temperature.

$$K_V = [(c_{11} + c_{22} + c_{33}) + 2(c_{12} + c_{13} + c_{23})] / 9 \quad \text{(Eq. S6)}$$

$$G_V = [(c_{11} + c_{22} + c_{33}) - (c_{12} + c_{13} + c_{23}) + 3(c_{44} + c_{55} + c_{66})] / 15 \quad \text{(Eq. S7)}$$

$$K_R = [(s_{11} + s_{22} + s_{33}) + 2(s_{12} + s_{13} + s_{23})]^{-1} \quad \text{(Eq. S8)}$$

$$G_R = 15 / [4(s_{11} + s_{22} + s_{33}) - 4(s_{12} + s_{13} + s_{23}) + 3(s_{44} + s_{55} + s_{66})] \quad \text{(Eq. S9)}$$

$$K_{VRH} = (K_V + K_R) / 2 \quad \text{(Eq. S10)}$$

$$G_{VRH} = (G_V + G_R) / 2 \quad \text{(Eq. S11)}$$

$$E = 9KG / (3K + G) \quad \text{(Eq. S12)}$$

$$\eta = (3K - 2G) / (6K + 2G) \quad \text{(Eq. S13)}$$

$$V_P = \sqrt{[(K_{VRH} + (4/3)G_{VRH}) / \rho]} \quad \text{(Eq. S14)}$$

$$V_S = \sqrt{[G_{VRH} / \rho]} \quad \text{(Eq. S15)}$$

The importance and effort involved in calculating these quantities connects directly with the discussion in the following section, relating to the possible occurrence of MS9 as a 'rock-forming' mineral in planetary interiors. The values that cannot be obtained simply by hydrostatic compression, those that manifest some aspect of the material's resistance to shear, are of high importance in modelling the viscoelastic response of planetary lithospheres to various time-dependent sources of stress. For many icy planetary bodies in the outer solar system, decoupling of the lithosphere from the deep interior by a liquid water (or brine) ocean acts to concentrate stress due to tidal forcing, de-spinning and shell re-orientation with respect to the interior (Nimmo & Schenk, 2006: Nimmo & Matsuyama, 2007: Wahr *et al.*, 2009: Geruo *et al.*, 2014. Another source of time-dependent stress where calculation of the viscoelastic strain requires quantities such as the shear modulus and the Poisson ratio is the secular cooling and freezing of subsurface oceans, which leads to extensional fracturing and normal-faulting on most icy worlds (Nimmo, 2004) or compressional orogeny on Titan (Cook-Hallet *et al.*, 2015).

Finally, the future deployment of seismometers on the surfaces of icy bodies – attached to vehicles such as the proposed *Laplace-P* Ganymede soft-lander or some kind of hardened penetrator (Gowan *et al.*, 2011) – will require an accurate database of V_P and V_S for constituent minerals in order to obtain a meaningful interpretation of the internal structure and to also apply seismic observations for characterisation of the short-term impact flux (Tsuji & Teanby, 2016).

Supplementary figures

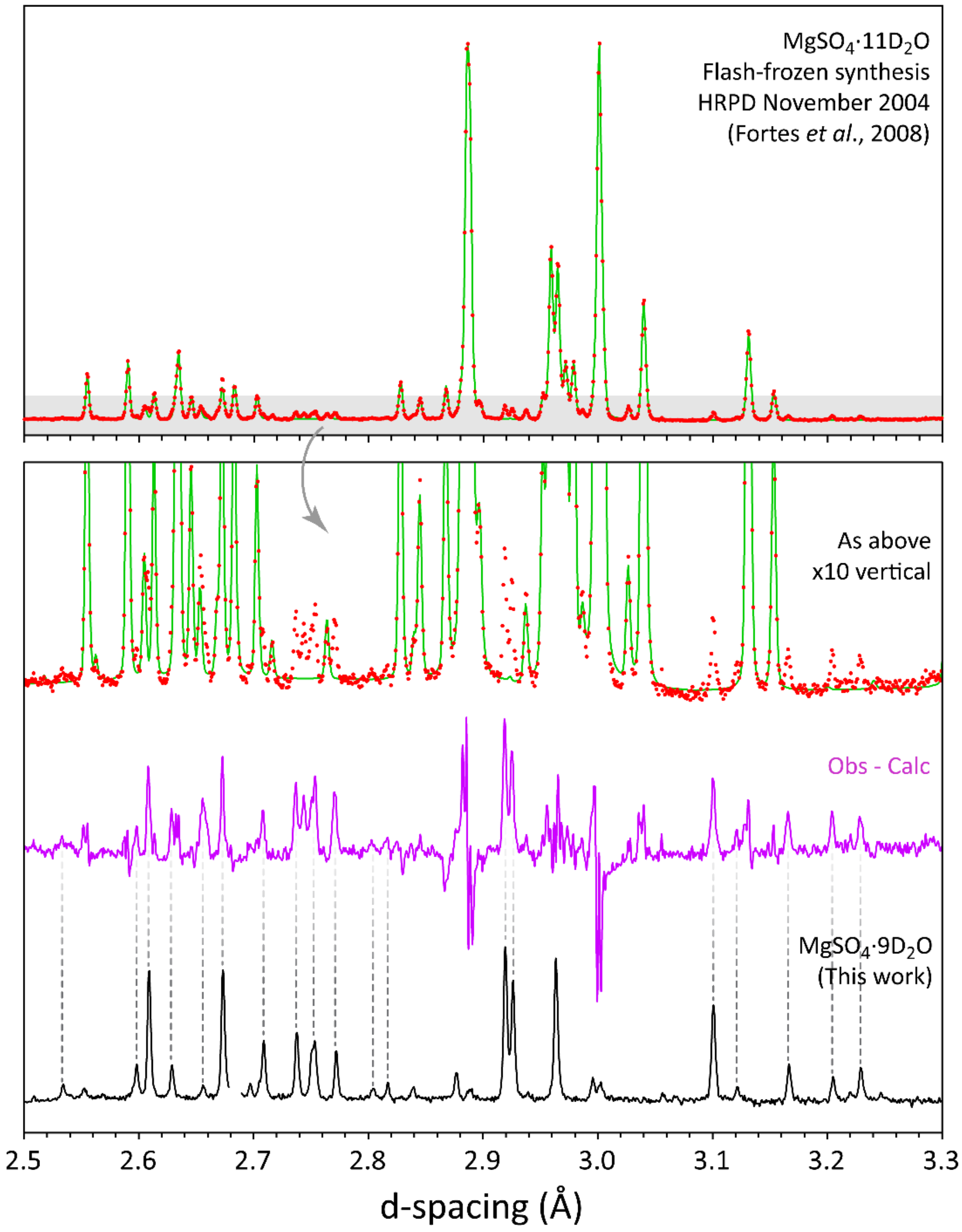

Figure S1:

Neutron powder diffraction data obtained on HRPD at ISIS in 2004 from $MgSO_4 \cdot 11D_2O$ revealed numerous parasitic peaks in the difference profile (purple, middle). At the time, we were unable to match these to any known phase of $MgSO_4 \cdot nD_2O$ (Fortes *et al.* 2008). It is now clear that these Bragg peaks are due to a small quantity of $MgSO_4 \cdot 9D_2O$. The black profile at the bottom shows the neutron powder data obtained from $MgSO_4 \cdot 9D_2O$ with a single peak from water ice omitted (near d-spacing = 2.65 Å); obvious matches between the black profile and unindexed peaks in the purple difference curve are highlighted with dashed lines. In places, intense overlapping peaks from $MgSO_4 \cdot 11D_2O$ make it impossible to identify peaks from $MgSO_4 \cdot 9D_2O$ (e.g., around 2.96 Å).

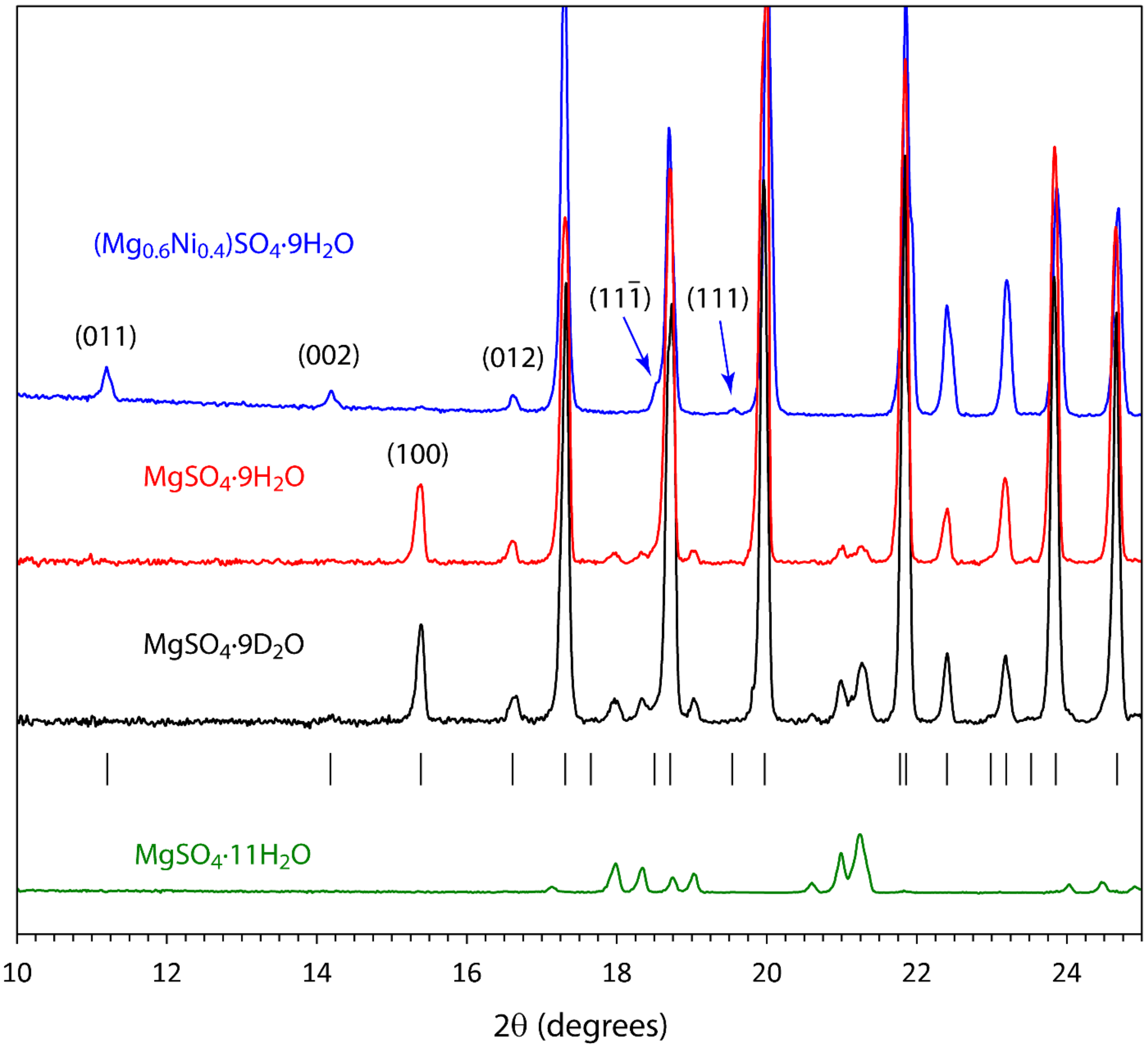

Figure S2

Low-angle region of the X-ray powder diffraction pattern (λ = 1.788996 Å) from cation-pure $MgSO_4·9H_2O$, $MgSO_4·9D_2O$ and $(Mg_{0.6}Ni_{0.4})SO_4·H_2O$, highlighting intensity differences in some low Miller-index Bragg peaks that were interpreted as being due to cation site ordering. Both of the cation-pure MS9 samples contain some MS11 and so a suitably scaled powder diffraction pattern of MS11 is given underneath the plots to indicate where the strongest peaks from this contaminant occur. Tick marks show the locations of reflections from MS9.

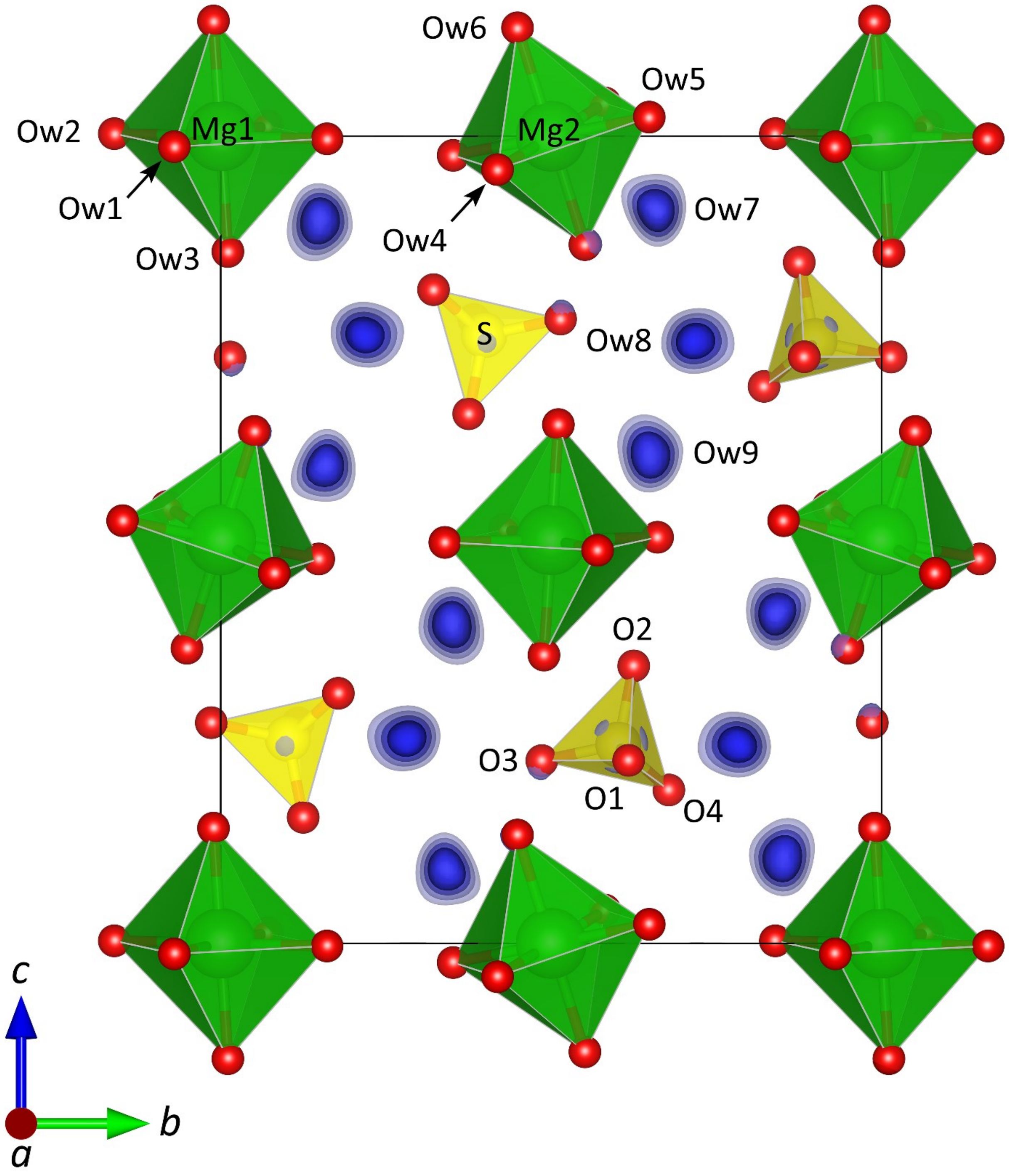

Figure S3

Trial structure for MS9 obtained from FOX in space-group *Pc* with the interstitial water molecules omitted. A difference Fourier synthesis phased on this partial structure reveals three 'blobs' of scattering density per formula unit, which correspond to the three water molecules necessary to confirm that this compound is an enneahydrate.

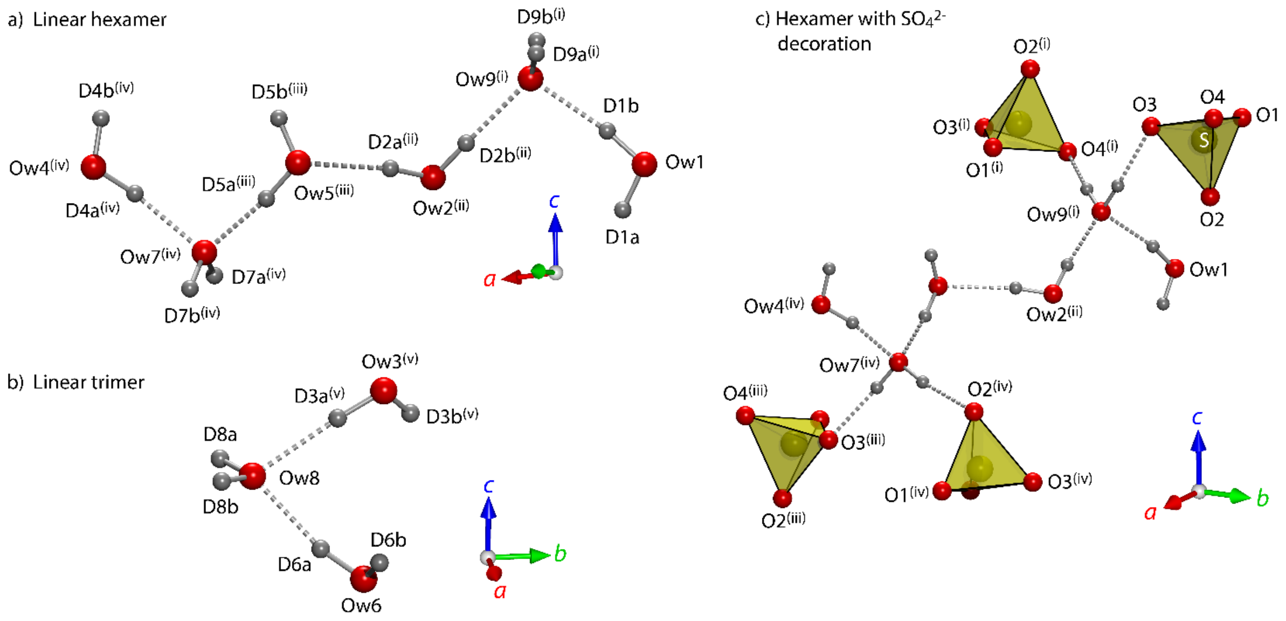

Figure S4

Illustration of the two water polymer structures in MS9, (a) the linear hexamer chain and (b) the small trimer. The cross-linking of sulfate tetrahedra through Ow7 and Ow9, which forms part of the hexameric chain, is shown in (c). Symmetry codes are same for each part of the figure: (*i*) [1−*x*, *y*−½, ½−*z*]; (*ii*) [1−*x*, −*y*, −*z*]; (*iii*) [1−*x*, *y*−1, *z*]; (*iv*) [2−*x*, −*y*, −*z*];
(*v*) [−*x*, ½+*y*, ½−*z*].

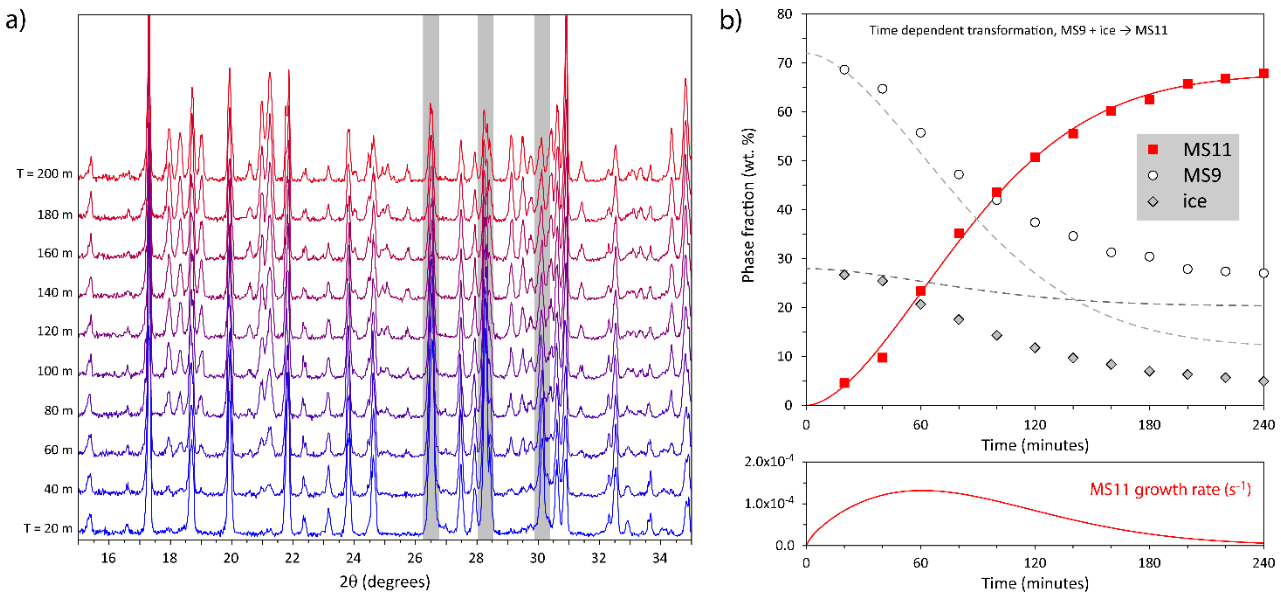

Figure S5

(a) Stack-plot of X-ray powder diffraction patterns (λ = 1.788996 Å) acquired as a function of time at T = 252 K showing the gradual transformation of MS9 to MS11. Grey vertical bars indicate the locations of the three strongest Bragg peaks from water ice.

(b) Refined phase fractions from the data shown in (a). The solid line through the symbols for MS11 reports the fit of Eq. 5 to the data. The two dashed lines show the expected phase fractions of ice and MS9 concomitant with the observed abundance of MS11 as a function of time.

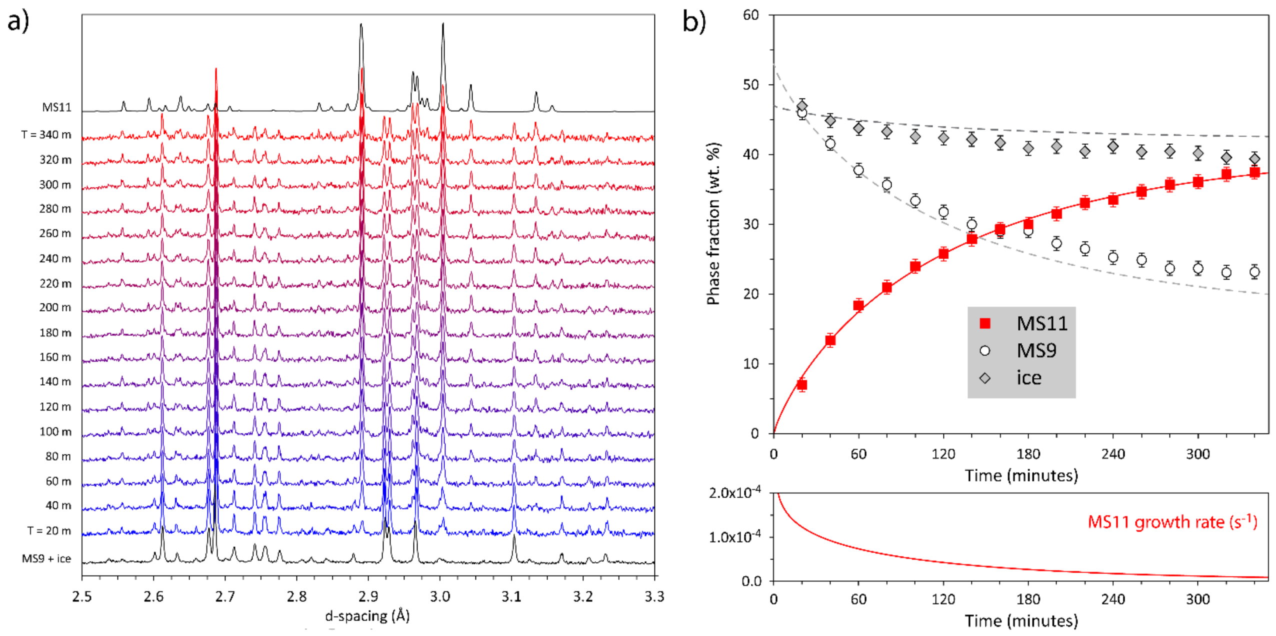

Figure S6

(a) Stack-plot of neutron powder diffraction patterns acquired as a function of time at T = 260 K showing the gradual transformation of MS9 to MS11 (Series 3).

(b) Refined phase fractions from the data shown in (a). The solid line through the symbols for MS11 reports the fit of Eq. 5 to the data. As in Figure S5 the dashed lines show the expected phase fractions of ice and MS9 concomitant with the observed abundance of MS11 as a function of time.

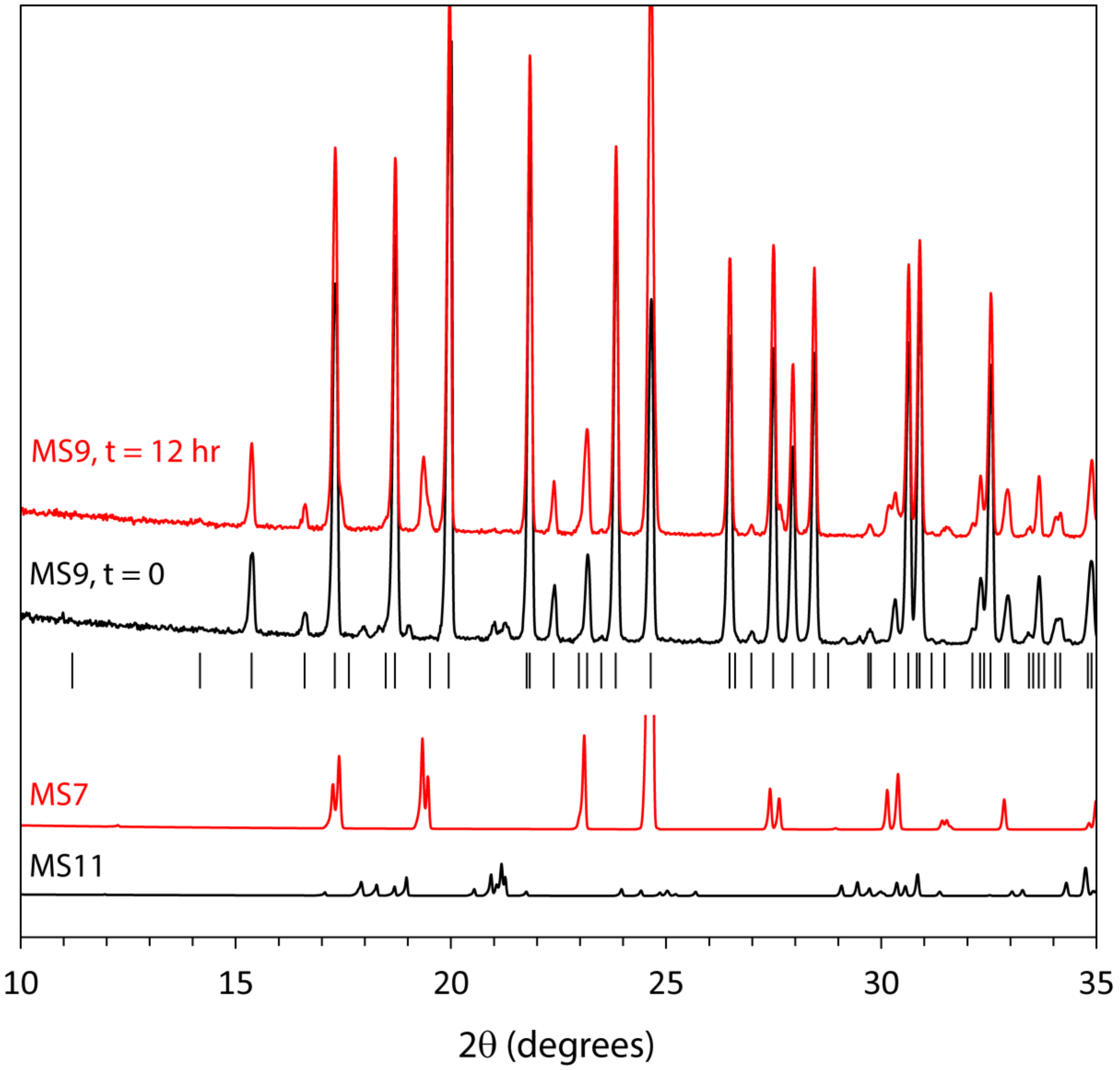

Figure S7

X-ray powder diffraction patterns (λ = 1.788996 Å) obtained by flash-freezing of liquids with close to stoichiometric 9-hydrate compositions. The black curve labelled "MS9, t = 0" corresponds to a 12 hr measurement at 252 K from an ice-free sample of MS9; small amounts of MS11 are present. The red curve labelled "MS9, t = 12 hr" shows a 7.5 hr dataset acquired directly after the first run had ended; the signature of MS11 is now absent and is replaced with MS7 (epsomite). The two profiles at the bottom show calculated patterns of MS7 and MS11 for visual comparison with the accessory phases in the MS9 data.

Supplementary tables

Table S1

Cell parameters of MS9 as a function of temperature. Uncertainties in T are ~ 0.05 K.

Series 1					
Temperature (K)	*a* (Å)	*b* (Å)	*c* (Å)	β (º)	V (Å^3)
9	6.7273(2)	11.9040(2)	14.6405(3)	95.199(1)	1167.61(2)
15	6.7274(2)	11.9035(2)	14.6403(4)	95.198(2)	1167.57(3)
20	6.7272(2)	11.9042(2)	14.6402(4)	95.199(2)	1167.60(3)
25	6.7274(2)	11.9039(2)	14.6404(4)	95.197(2)	1167.61(3)
30	6.7274(2)	11.9043(2)	14.6407(4)	95.197(2)	1167.68(3)
35	6.7276(2)	11.9045(2)	14.6404(4)	95.196(2)	1167.70(3)
40	6.7275(2)	11.9048(2)	14.6405(4)	95.196(2)	1167.73(3)
45	6.7278(2)	11.9052(2)	14.6401(4)	95.194(2)	1167.79(3)
50	6.7279(2)	11.9056(2)	14.6400(4)	95.194(2)	1167.84(3)
55	6.7282(2)	11.9065(2)	14.6406(4)	95.193(2)	1168.04(3)
60	6.7283(2)	11.9070(2)	14.6408(4)	95.191(2)	1168.13(3)
65	6.7288(2)	11.9081(2)	14.6404(4)	95.192(2)	1168.28(3)
70	6.7293(2)	11.9089(2)	14.6404(4)	95.189(2)	1168.45(3)
75	6.7297(2)	11.9096(2)	14.6417(4)	95.188(2)	1168.70(3)
80	6.7302(2)	11.9107(2)	14.6418(4)	95.186(2)	1168.90(3)
85	6.7310(2)	11.9118(2)	14.6416(4)	95.183(2)	1169.14(3)
90	6.7314(2)	11.9131(2)	14.6426(4)	95.183(2)	1169.41(3)
95	6.7320(2)	11.9141(2)	14.6432(4)	95.182(2)	1169.68(3)
100	6.7329(2)	11.9153(2)	14.6434(4)	95.181(2)	1169.96(3)
105	6.7337(2)	11.9170(2)	14.6435(4)	95.183(2)	1170.27(3)
110	6.7344(2)	11.9183(2)	14.6443(4)	95.179(2)	1170.60(3)
115	6.7352(2)	11.9194(2)	14.6462(3)	95.178(1)	1170.99(3)
120	6.7361(1)	11.9209(2)	14.6466(3)	95.178(1)	1171.32(3)
125	6.7371(1)	11.9225(2)	14.6470(3)	95.174(1)	1171.69(3)
130	6.7377(1)	11.9241(2)	14.6487(3)	95.177(1)	1172.10(3)
135	6.7389(1)	11.9258(2)	14.6495(3)	95.172(1)	1172.53(3)
140	6.7400(1)	11.9274(2)	14.6499(3)	95.171(1)	1172.93(3)
145	6.7411(1)	11.9292(2)	14.6510(3)	95.170(1)	1173.39(3)
150	6.7420(1)	11.9310(2)	14.6522(3)	95.168(1)	1173.83(3)
155	6.7431(1)	11.9330(2)	14.6536(3)	95.170(1)	1174.32(3)
160	6.7442(1)	11.9347(2)	14.6539(3)	95.167(1)	1174.70(3)
165	6.7454(1)	11.9370(2)	14.6559(3)	95.167(1)	1175.29(3)
170	6.7466(1)	11.9384(2)	14.6570(3)	95.165(1)	1175.72(3)
175	6.7478(1)	11.9405(2)	14.6583(3)	95.164(1)	1176.24(3)
180	6.7489(1)	11.9429(2)	14.6594(3)	95.163(1)	1176.77(3)
185	6.7502(1)	11.9450(2)	14.6611(3)	95.163(1)	1177.33(2)
190	6.7515(1)	11.9471(2)	14.6624(3)	95.165(1)	1177.88(2)
195	6.7528(1)	11.9491(2)	14.6635(3)	95.161(1)	1178.40(2)
200	6.7541(1)	11.9513(2)	14.6651(3)	95.159(1)	1178.97(2)
205	6.7554(1)	11.9538(2)	14.6665(3)	95.160(1)	1179.57(2)
210	6.7569(1)	11.9561(2)	14.6680(3)	95.159(1)	1180.17(2)
215	6.7582(1)	11.9582(2)	14.6695(3)	95.158(1)	1180.74(2)
220	6.7595(1)	11.9606(2)	14.6709(2)	95.158(1)	1181.31(2)
225	6.7610(1)	11.9632(2)	14.6721(2)	95.156(1)	1181.93(2)
230	6.7624(1)	11.9647(1)	14.6741(2)	95.154(1)	1182.49(2)

Series 2					
Temperature (K)	*a* (Å)	*b* (Å)	*c* (Å)	β (º)	V (Å^3)
220	6.76019(8)	11.9635(1)	14.6703(2)	95.154(1)	1181.67(2)
225	6.76170(9)	11.9643(1)	14.6718(2)	95.152(1)	1182.14(2)
230	6.76317(9)	11.9660(1)	14.6733(2)	95.151(1)	1182.68(2)
235	6.76478(9)	11.9682(1)	14.6748(2)	95.151(1)	1183.30(2)
240	6.76651(9)	11.9707(1)	14.6761(2)	95.149(1)	1183.96(2)
245	6.76821(9)	11.9730(1)	14.6774(2)	95.147(1)	1184.61(2)
250	6.76995(9)	11.9756(1)	14.6787(2)	95.146(1)	1185.27(2)
255	6.7718(1)	11.9782(1)	14.6797(2)	95.144(1)	1185.94(2)
260	6.7735(1)	11.9808(2)	14.6808(2)	95.142(1)	1186.59(2)
Series 3					
Temperature (K)	*a* (Å)	*b* (Å)	*c* (Å)	β (º)	V (Å^3)
260	6.7743(1)	11.9828(2)	14.6827(3)	95.143(1)	1187.06(3)
250	6.7706(1)	11.9772(2)	14.6779(3)	95.148(1)	1185.47(3)
220	6.7605(1)	11.9659(2)	14.6692(3)	95.159(1)	1181.86(2)
210	6.7575(1)	11.9640(2)	14.6659(2)	95.157(1)	1180.89(2)
200	6.7549(1)	11.9604(2)	14.6627(2)	95.159(1)	1179.83(2)
190	6.7523(1)	11.9561(2)	14.6601(2)	95.161(1)	1178.73(2)
180	6.7496(1)	11.9517(2)	14.6573(3)	95.163(1)	1177.60(2)
170	6.7468(1)	11.9471(2)	14.6550(3)	95.165(1)	1176.47(2)
160	6.7444(1)	11.9428(2)	14.6523(3)	95.166(1)	1175.41(2)
150	6.7420(1)	11.9388(2)	14.6504(3)	95.171(1)	1174.43(3)
140	6.7397(1)	11.9352(2)	14.6484(3)	95.173(1)	1173.51(2)
130	6.7376(1)	11.9316(2)	14.6464(3)	95.174(1)	1172.64(2)
120	6.7356(1)	11.9286(2)	14.6450(3)	95.178(1)	1171.87(2)
110	6.7337(1)	11.9254(2)	14.6437(4)	95.179(1)	1171.12(2)
100	6.7322(1)	11.9222(2)	14.6423(4)	95.183(1)	1170.43(3)
90	6.7307(1)	11.9198(2)	14.6407(4)	95.185(1)	1169.81(3)
80	6.7295(1)	11.9174(2)	14.6401(4)	95.189(1)	1169.30(3)
70	6.7283(1)	11.9157(2)	14.6399(4)	95.190(1)	1168.91(3)
60	6.7278(1)	11.9137(2)	14.6386(4)	95.194(1)	1168.51(3)
50	6.7269(1)	11.9126(2)	14.6398(4)	95.196(1)	1168.33(3)
40	6.7269(1)	11.9114(2)	14.6387(4)	95.198(1)	1168.13(3)
30	6.7266(1)	11.9105(2)	14.6392(4)	95.199(1)	1168.02(3)
20	6.7264(1)	11.9105(2)	14.6395(4)	95.203(1)	1168.01(3)
9	6.7264(1)	11.9105(2)	14.6397(3)	95.202(1)	1168.03(2)

Table S2

Cell parameters of MS9 as a function of pressure along the 240 K isotherm. Uncertainties in T are ~ 0.5 K for the first six points and ~ 1 K for the final point. Uncertainties in P are 0.3 % for the first six points, measured in a gas-pressure vessel, and 2.25 % for the final point measured in a Paris-Edinburgh press.

Series 4					
Pressure (MPa)	*a* (Å)	*b* (Å)	*c* (Å)	β (°)	V (Å^3)
10.0	6.7676(9)	11.967(2)	14.679(2)	95.12(1)	1184.1(2)
97.3	6.7584(7)	11.943(1)	14.669(2)	95.17(1)	1179.2(1)
205.3	6.7452(6)	11.911(1)	14.658(2)	95.222(6)	1172.7(1)
295.5	6.7348(5)	11.885(1)	14.646(1)	95.266(6)	1167.3(1)
417.6	6.7218(6)	11.851(1)	14.631(2)	95.320(6)	1160.5(1)
542.5	6.7095(4)	11.820(1)	14.615(1)	95.366(5)	1154.0(1)
1067(24)*	6.667(1)	11.690(2)	14.530(3)	95.50(1)	1127.2(2)

*From a separate measurement described in Fortes *et al.* (submitted paper FX5002).

Table S3

Debye model fit parameters.

	Single-Debye model	Double-Debye model	Double-Debye equivalent of Einstein model
V_0 (cm^3 mol^{-1})	175.806(4)	175.7872*	175.7872*
V_0 (Å^3)	1167.73(3)	1167.6059*	1167.6059*
Q (J cm^{-3})	671(6)x10^4	342.8x10^4*	342.8x10^4*
K_0/γ (GPa)	38(1)	19.50*	19.50*
θ_1^D (K)	449 ± 7	323 ± 9	351 ± 1
θ_2^D (K)	–	904 ± 59	1488 ± 20
x	1†	0.30(2)	0.359(2)
y	–	0.34(1)	0.59(1)

*Parameter fixed during fitting

†By definition

Table S4

Elastic stiffnesses, c_{ij}, and elastic compliances, s_{ij}, of MS9 from DFT calculations (athermal, WC GGA) compared with the measured elastic constants of various orthorhombic and monoclinic heptahydrates of divalent metal sulfates.

	MS9 (this work)		MS7*	NiS7*	ZnS7*	CoS7*	FeS7*
ij	c_{ij} (GPa)	s_{ij} (GPa^{-1})	c_{ij} (GPa)	c_{ij} (GPa)	c_{ij} (GPa)	c_{ij} (GPa)	c_{ij} (GPa)
11	39.2(8)	0.037(1)	32.5	35.3	33.2	33.5	34.9
22	36.3(8)	0.037(1)	28.8	31.1	29.3	37.8	37.6
33	53.6(8)	0.0288(7)	31.5	33.5	32.0	37.1	36.0
44	14.5(4)	0.071(2)	7.8	9.1	7.8	6.0	6.4
55	8.4(3)	0.124(5)	15.6	17.2	15.3	5.8	5.6
66	8.4(2)	0.123(3)	9.0	9.9	8.3	10.1	9.6
12	15.4(3)	−0.0086(6)	17.4	19.8	17.2	20.5	20.8
13	23.0(7)	−0.0131(9)	18.2	20.1	20.0	15.8	17.4
15	0.72(6)	−0.0095(6)	–	–	–	1.6	−2.0
23	20.3(5)	−0.0101(7)	18.2	20.1	19.8	15.8	17.2
25	−1.09(9)	0.0015(5)	–	–	–	−1.8	−1.9
35	−3.34(2)	0.0112(5)	–	–	–	−4.7	−1.4
46	−1.95(8)	0.016(1)	–	–	–	1.6	0.1

*Alexandrov *et al.* (1963)

Table S5

Derived 'bulk' elastic properties calculated from the stiffnesses and compliances given in Table S3 (*cf.*, equations 13 – 22).

	MS9 (this work)	MS7*	NiS7*	ZnS7*	CoS7*	FeS7*
K_V (GPa)	27.4	22.3	24.4	23.2	23.6	24.4
K_R (GPa)	25.8	22.2	24.4	23.0	22.9	23.7
K_{VRH} (GPa)	26.6	22.2	24.4	23.1	23.3	24.1
G_V (GPa)	11.0	9.1	9.9	8.8	8.1	7.9
G_R (GPa)	10.1	8.1	8.7	7.8	6.7	7.4
G_{VRH} (GPa)	10.5	8.6	9.3	8.3	7.4	7.6
E (GPa)	27.9	22.9	24.8	22.2	20.1	20.7
η	0.325	0.328	0.331	0.340	0.356	0.357
V_P (m s^{-1})	5024	4479	4347	4162	4126	4247
V_S (m s^{-1})	2556	2264	2187	2050	1951	2006

*Alexandrov *et al.* (1963)

Supplementary references

Alexandrov. K. S., Rhyzhova. T. V., & Rostuntseva, A. I. (1963). *Soviet Phys. Cryst.* **7**, 753–755. [Translated from, *Kristallographiya* **7**, 930–933 (1962)].

Arbeck, D., Haussühl, E., Bayarjagal, L., Winkler, B., Paulsen, N., Haussühl, S., & Milman, V. (2010). *Eur. Phys. J. B* **73**, 167–175.

Cook-Hallett, C., Barnes, J. W., Kattenhorn, S. A., Hurford, T., Radebaugh, J., Stiles, B., & Beuthe, M. (2015). *J. Geophys. Res. Planets*, **120**, 1220–1236. doi:10.1002/2014JE004645.

Fortes, A. D., Wood, I. G., Brodholt, J. P., Alfredsson, M., Vocadlo, L., McGrady, G. S. & Knight, K. S. (2003). *J. Chem. Phys.* **119**, 10806–10813.

Geruo, A., Wahr, J., & Zhong, S. (2014). *J. Geophys. Res. Planets* **119**, 659–678.

Gowen, R. A., and 46 co-authors (2011). *Adv. Space Res.* **48**, 725–742.

Hill, R. (1952). *Proc. Phys. Soc. London*. **65**, 349–354.

Nimmo, F. (2004). *J. Geophys. Res.* **109**, E12001, doi:10.1029/2004JE002347.

Nimmo, F., & Matsuyama, I. (2007). *Geophys. Res. Lett.* **34**, L19203, doi:10.1029/2007GL030798.

Nimmo, F., & Schenk, P. (2006). *J. Struct. Geol.* **28**, 2194–2203.

Reuss, A. (1929). *Z. Angew. Math. Mech*. **9**, 49–58.

Tsuji, D., & Teanby, N. A. (2016). *Icarus* **277**, 39–55.

Voigt, W. (1910). *Lehrbuch der Kristallphysik*, Taubner, Leipzig.

Wahr, J., Selvans, Z. A., Mullen, M. E., Barr, A. C., Collins, G. C., Selvans, M. M., & Pappalardo, R. T. (2009). *Icarus* **200**, 188–206.

END OF ELECTRONIC SUPPLEMENT
